# Mortality Among People With Attention-Deficit/Hyperactivity Disorder: A Systematic Review and Meta-Analysis

**DOI:** 10.1001/jamanetworkopen.2026.39448

**Published:** 2026-10-19

**Authors:** Lucy Corrigan, Lauren Heywood, Gavin R. Stewart, Elizabeth O’Nions, Henry Shelford, Joshua Stott, Amber John

**Affiliations:** 1 Department of Psychology, University of Liverpool, Liverpool, United Kingdom; 2 Social, Genetic, and Developmental Psychiatry Centre, Kings College London, London, United Kingdom; 3 Bradford Institute for Health Research, Bradford Teaching Hospitals NHS Foundation Trust, Bradford, United Kingdom; 4 ADHD UK, London, United Kingdom; 5 Clinical, Educational, and Health Psychology, University College London, London, United Kingdom

## Abstract

**Importance:**

Attention-deficit/hyperactivity disorder (ADHD) is associated with adverse life outcomes and increased mortality. Clarifying the magnitude of mortality risks using international data and the potential association between medication and mortality is essential to inform clinical care and motivate public health strategies to address mortality.

**Objectives:**

To examine the association between ADHD and all-cause and cause-specific mortality and to assess whether mortality risk differs according to ADHD medication use.

**Data Sources:**

A comprehensive search of PubMed, Web of Science, PsycINFO, EMBASE, MEDLINE, and CINAHL was conducted from database inception to July 8, 2025.

**Study Selection:**

Eligible studies included quantitative research across all age groups reporting all-cause and/or cause-specific mortality in individuals with ADHD, defined via diagnostic criteria, self-identification, trait measures, or ADHD polygenic risk scores. Exclusion criteria were nonoriginal research, qualitative studies, non–peer-reviewed publications, and studies not reporting mortality outcomes.

**Data Extraction and Synthesis:**

Data extraction was conducted independently by 2 reviewers. Study quality was assessed by 1 reviewer. Summary data were extracted from published reports, and pooled hazard ratios (HRs) were calculated using random-effects model meta-analysis.

**Main Outcomes and Measures:**

The primary outcomes were all-cause mortality and cause-specific mortality (suicide, unintentional or intentional injury, and natural causes), measured using HRs with 95% CIs.

**Results:**

Nineteen studies including 15 920 970 participants (36.1% female) were included. ADHD was associated with increased all-cause mortality (HR, 1.71; 95% CI, 1.31-2.24; *P* < .001). Significant associations were also observed for suicide (HR, 1.89; 95% CI, 1.21-2.95; *P* = .005), unintentional or intentional injury (HR, 1.55; 95% CI, 1.22-2.00; *P* < .001), and natural-cause mortality (HR, 1.27; 95% CI, 1.16-1.38; *P* < .001). ADHD medication use in people with ADHD was associated with reduced all-cause mortality (HR, 0.75; 95% CI, 0.64-0.87; *P* < .001). Substantial heterogeneity was observed across studies.

**Conclusions and Relevance:**

In this systematic review and meta-analysis of ADHD and mortality, ADHD was associated with increased mortality across multiple causes; and ADHD medication was associated with a decreased all-cause mortality risk, underscoring the importance of timely diagnosis and treatment. However, the heterogeneity observed across studies highlights the need for standardized methods and improved control of confounding factors to better characterize mortality risk among individuals with ADHD.

## Key Points

**Question:**

Is there an association between attention-deficit/hyperactivity disorder (ADHD) and ADHD medication and all-cause and cause-specific mortality (suicide, unintentional injury, and natural cause)?

**Findings:**

In this systematic review and meta-analysis of 19 studies with more than 15.9 million participants, ADHD was associated with increased all-cause mortality, suicide mortality, unintentional or intentional injury mortality, and natural-cause mortality, whereas ADHD medication use was associated with reduced all-cause mortality.

**Meaning:**

These results suggest that people with ADHD have increased risk of mortality across all-cause, suicide, unintentional or intentional injury, and natural cause; and ADHD medication may reduce the risk of all-cause mortality among people with ADHD, underscoring importance of timely diagnosis and treatment.

## Teaser

This systematic review and meta-analysis examines the association between attention-deficit/hyperactivity disorder (ADHD) and all-cause and cause-specific mortality and medication use.

## Introduction

Attention-deficit/hyperactivity disorder (ADHD) is a neurodevelopmental condition characterized by persistent inattention, hyperactivity, and/or impulsivity, which occurs in multiple contexts and interferes with daily functioning.^1^ It is a heterogenous condition, with diverse symptom presentations and severity.^2^ ADHD is widely recognized for its impact on educational, occupational, and psychosocial functioning.^3^ Although ADHD was previously thought to mostly affect children, more recently, it has been recognized as frequently persisting across the lifespan,^4^ with many individuals continuing to display traits of ADHD into older adulthood. In the context of a globally aging population, understanding long-term outcomes, such as mortality, is increasingly important to motivate systemic attempts to improve the health of people with ADHD. In line with this there is increasing evidence that ADHD may also be associated with adverse long-term health outcomes, including premature mortality.^5^ With some work pointing to a 6- to 7-year life expectancy gap between people with ADHD and those without.^5^

Increased mortality risks in the ADHD population may occur through multiple plausible pathways. For example, core traits of ADHD (such as differences in impulse control, emotional regulation, and risk-taking behaviors) may increase the risk of mortality through increased unintentional or intentional injuries and increased co-occurring mental health conditions, such as depression in people with ADHD,^6^ also increasing the risk of suicide and consequent mortality in this population. In addition, risk of natural-cause mortality may also be increased in ADHD through indirect mechanisms, including (1) health risk behaviors more common in people with ADHD (eg, increased smoking and alcohol use),^7^ (2) challenges with engagement and access to health care services,^8^ and (3) higher rates of co-occurring physical health conditions, such as cardiovascular disease and metabolic disorders.^9,10^ It is important to recognize that dominant societal attitudes often locate problems such as substance use within the individual, framing them as disorders. However, this perspective can obscure the role of structural inequalities, unmet needs, and experiences of exclusion. For many individuals, particularly those who are neurodivergent, substance use may function as a response to environments that present significant barriers to accessing appropriate and inclusive support.

Despite this, the current evidence base is heterogenous, with variation in study design, populations, and outcome definitions (for cause-specific mortality), making it difficult to draw conclusions about the magnitude of the risk of mortality in ADHD. There is consequent need for a systematic review and quantitative synthesis to support understanding of the degree of increased mortality risk experienced by people with ADHD. To our knowledge, this has not yet been done, and the only previous systematic review^11^ conducted examined mortality in neurodevelopmental conditions more broadly, including reviews that combine clinical populations such as ADHD with autism, which could obscure condition-specific patterns of risk because although ADHD and autism commonly co-occur,^12^ they differ in their presentations, patterns of co-occurring health conditions, mechanisms of risk, and life course trajectories.^13,14^ This may result in distinct mortality risks and causes of death. In addition, the original study used diagnostic criteria to determine eligibility for inclusion. In contrast, this review included studies using both trait measures and self-identification to better reflect the general clinical population, given the variation in diagnostic practices and trends. Furthermore, since this review,^12^ additional studies have been published contributing new evidence and updating the overall understanding of mortality risk in ADHD. The emergence of additional studies also provides an opportunity to examine mortality across specific causes of death and potential modifiers of risk, including ADHD medication use, which was not assessed in the previous meta-analysis.^12^ As such, the strength of mortality risk as well as how risk differs across cause-specific mortality, such as suicide, unintentional or intentional injury, and natural causes, remain underexplored.

Many people with ADHD take prescribed medications. Global trends suggest a steady increase in the prevalence of ADHD medication use.^15^ Specifically, in England, the number of people obtaining central nervous system stimulants and medicines for ADHD has more than doubled during the past 6 years, with approximately 278 000 receiving medications in 2023 to 2024, with a particular acceleration in the past 2 years.^16^ These statistics highlight the increasing recognition and treatment of ADHD. Evidence as to the potential role of ADHD medication in moderating mortality risk in ADHD suggests an adverse impact on cardiovascular outcomes^4^ but a positive impact on other potential routes to mortality, including reduced risks of various negative outcomes, such as suicidal behavior, substance use, transport accidents, and criminality.^17^ Despite this, current evidence on whether medication use in people with ADHD is associated with a change in mortality risk is conflicting.

This systematic review and meta-analysis aims to synthesize the existing literature on mortality in individuals with ADHD to update prior work^12^ on the topic with data spanning from 1990 to 2019. The aims of this review are to examine whether ADHD is associated with higher all-cause mortality compared with people without ADHD, whether ADHD is associated with cause-specific mortality (suicide, death due to unintentional or intentional injury, or natural-cause death) compared with people without ADHD, and whether ADHD medication use in people with ADHD is associated with reduced all-cause mortality.

## Methods

### Systematic Search and Study Selection

This systematic review and meta-analysis followed the Preferred Reporting Items for Systematic Reviews and Meta-Analyses (PRISMA) reporting guideline.^18^ Relevant studies were identified through a systematic and comprehensive search of 6 electronic databases (PubMed, Web of Science, PsycINFO, EMBASE, MEDLINE, and CINAHL) on July 8, 2025. A list of search terms was generated and tested to identify relevant studies. To develop search terms, we performed an initial exploratory phase that involved reviewing strategies and search terms used in published systematic reviews^11,19^ to investigate similar populations and/or outcomes. The relevant terms identified were used to inform the strategy for the current review. Search terms were then piloted by the research team to ensure they were identifying the relevant articles. Search terms related to ADHD and mortality (eFigure 1 in Supplement 1). No language, time, or other limits were applied to the search strategy. Reference lists of all studies that met the inclusion criteria were searched based on the same criteria.

Relevant studies identified from the search were imported into Rayyan.ai. Duplicate articles were identified and resolved or removed. An author (L.C.) screened all articles by title and abstract (9363 articles). Another author (L.H.) blind screened 10% of all articles by title and abstract (936 articles), and interrater agreement was calculated. Studies were excluded from the review if they did not meet inclusion criteria. Full texts were screened (L.C.) against the same inclusion and exclusion criteria (165 articles), and 10% of full texts were additionally blind screened (L.H.). Conflicts were discussed and resolved among the authors. If full-text articles could not be retrieved, they were excluded at this stage.

Studies were eligible for inclusion if they included people with ADHD (defined using diagnostic criteria, self-identification, or ADHD trait measures) and reported mortality outcomes, including all-cause and/or cause-specific mortality. Quantitative studies across all age groups were included. Studies investigating associations between ADHD polygenic risk scores and mortality were also included. Studies were excluded if they did not report relevant mortality outcomes, did not examine ADHD as the primary indicator of mortality, or used qualitative methods. We also excluded systematic reviews, non–peer-reviewed studies, and publications that did not present original data (a full list of all excluded articles is given in eFigure 2 in Supplement 1).

We also included studies testing associations between ADHD medication use and mortality outcomes in people with ADHD. For these, studies were excluded if they were looking at associations between ADHD medication use and mortality in general population samples (not restricted to people with ADHD).

### Methodologic Quality Assessment

The Newcastle-Ottawa Scale^20^ was used to assess methodologic quality of both cohort and case-control studies. According to this checklist, studies are assessed on the quality of selection, comparability, and outcome and are scored from 0 to 9, with 0 indicating poor quality and 9 indicating good quality. Based on scoring, studies are marked as good, fair, or poor quality.

### Data Extraction

Two reviewers (L.C. and A.J.) independently extracted data on study characteristics and outcome measures. Differences were discussed and resolved among reviewers. The following data were extracted for each included article: article title, authors, year of publication, ADHD measure, sample size, means and SDs for age, and gender split in the sample. In addition, information regarding the study design, type of statistical analysis used, and variables was also extracted. All relevant statistical information was extracted from the included articles.

### Statistical Analysis

Studies were grouped for meta-analysis based on reported outcomes (all-cause mortality, suicide, death from unintentional or intentional injuries, and natural-cause death). Effect sizes were pooled in a random-effects model. The heterogeneity of each model was assessed using the *I*^2^ and *Q* statistic. The following cutoffs were referred to when interpreting *I*^2^ statistics: 0% to 25%, low heterogeneity; 25% to 50%, moderate heterogeneity; 50% to 75%, substantial heterogeneity; and 75% to 100%, high heterogeneity.^21^ The extent of publication bias was evaluated using the Egger test and the production of funnel plots. Two-sided *P* < .05 was considered as statistically significant. The analyses were performed using RStudio software, version 2025.05.0 + 496 and the metafor package for R (R Foundation for Statistical Computing).^22^

## Results

### Study Selection

A total of 20 034 articles were identified from the initial database search. After removal of duplicates and an initial title and abstract screen, 162 titles were identified for full-text screening. This resulted in 19 studies meeting the inclusion criteria for the current review.^5,23,24,25,26,27,28,29,30,31,32,33,34,35,36,37,38,39,40^ Twelve were included in the meta-analyses,^5,23,24,25,26,27,28,29,30,31,32,33^ and the remaining 7 are reported narratively.^34,35,36,37,39,40^ The flow diagram of study selection is presented in Figure 1.

**Figure 1.**
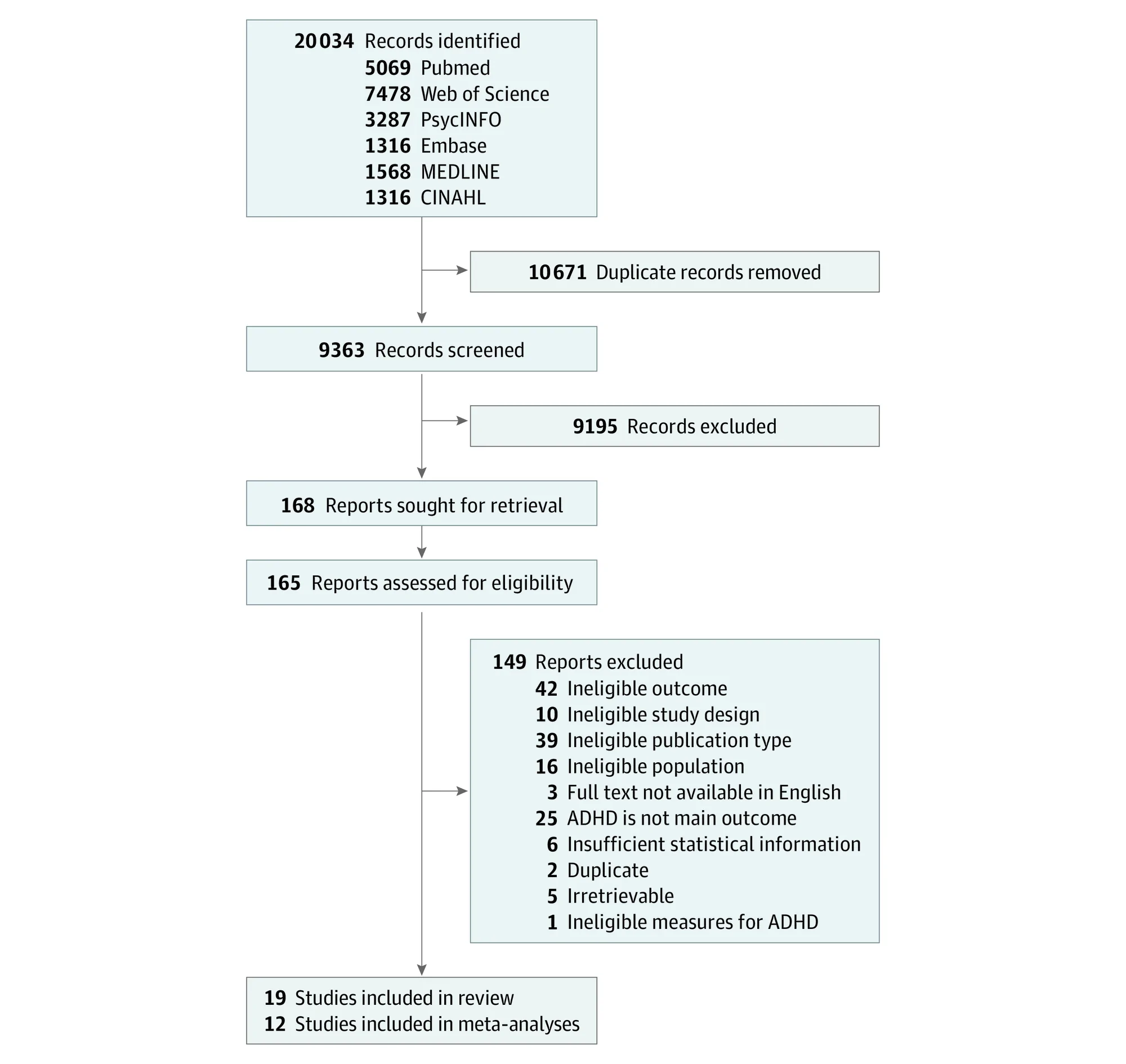
PRISMA Flow Diagram ADHD indicates attention-deficit/hyperactivity disorder.

### Characteristics of Studies

The characteristics of included studies are presented in the Table. The included studies were published between 2012 and 2025. Total sample sizes in the studies ranges from 839 to 2 924 780, with a total of 15 920 970 across all included studies. Four studies were conducted in the US,^34,35,37,39^ 3 in Canada,^29,32,38^ 3 in Sweden,^27,33,36^ 3 in Taiwan,^23,26,31^ 2 in the UK,^5,25^ 2 in Finland,^24,40^ and 2 in Denmark.^28,30^ All studies reported on mortality outcomes, with 8 outcomes in total: all-cause mortality, natural-cause mortality, suicide, unnatural-cause mortality, unintentional injury or accident, homicide, and/or complications due to COVID-19. Four of the included studies^25,34,36,37^ had participants composed of a greater female proportion (50.2%-54.5%), whereas the remaining 15 studies^5,23,24,26,27,28,29,30,31,32,33,35,38,39,40^ had participants of a greater male proportion (female proportions, 17.8%-49.0%). Sixteen studies^5,24,26,27,28,29,30,31,32,34,35,36,37,38,39,40^ used diagnostic codes to identify individuals with ADHD, 1 study^33^ used self-reported ADHD, 1 study^23^ used either diagnostic codes or trait measures, and 1 study^25^ used polygenic risk scores for ADHD. Of the 19 studies, 17 studies^5,24,25,26,27,28,29,30,31,32,33,34,35,36,37,38,39^ were given a good quality rating, 2 studies^23,40^ were given a fair quality rating, and none were given a poor rating.

**Table.** Demographics and Characteristics of the Included Studies

Study	Location	Setting	Age, mean (SD), y	Women, %	Outcomes assessed	ADHD diagnostic and inclusion criteria	Follow up or duration observed, y	No. in the analysis	Quality rating
O’nions et al,^5^ 2025	UK	UK electronic primary care heath records from IQVIA Medical Research Data	NR	22.17	All-cause mortality; years of life lost	Diagnostic code lists	Men: 2.54 y; women: 2.17 y	Total: 330 429; ADHD: 30 039; control: 300 390	Good
Jeng et al,^23^ 2024	Taiwan	Taiwan’s National Health Insurance Research Database	NR	21.92	All-cause mortality; natural cause mortality; unnatural cause mortality; accident mortality; suicide mortality	*ICD-9-CM* codes	Observation period: 2003-2017	Total: 1 169 430; ADHD: 233 886; control: 935 544	Fair
Schiavone et al,^24^ 2022	Finland	Perinatal risk cohort from a single maternity unit in Helsinki between 1971 and 1974	NR	28.70	Cause-specific mortality for natural and unnatural causes of death; all cause-mortality	Questionnaires and clinical assessments and *DSM-IV* codes	NR	Total: 839; ADHD: 115; control: 724	Good
Ajnakina et al,^25^ 2022	England	English longitudinal study of aging	64.7 (9.5)	53.8	All-cause mortality	Polygenic risk scores	79 829.8 Person-years	Total: 7133	Good
Chen et al,^26^ 2019	Taiwan	Cross-national Taiwanese registry	9.61 (5.74)	24.12	All-cause mortality; suicide; unintentional injury; homicide; natural cause	*ICD-9* code	15.1 million Person-years	Total: 2 207 840; ADHD: 275 980; control: 1 931 860	Good
Sun et al,^27^ 2019	Sweden	Swedish national registers with individuals born from 1983 to 2009	NR	33.17	All-cause mortality; suicide; natural cause; unintentional injury; unnatural cause	*ICD-10* code	29 237 993 Person-years	Total: 2 675 615; ADHD: 86 670; control: 2 588 945	Good
Dalsgaard et al,^28^ 2015	Denmark	Danish national registers following up individuals from first birthday to 2013	12.3 (6.1), age at diagnosis	26.44	All-cause mortality rate	*ICD-8* and *ICD-10* code	24.9 million Person-years	Total: 1 922 248; ADHD: 32 061; control: 1 890 187	Good
Diallo et al,^29^ 2021	Quebec	Quebec Integrated Chronic Disease Surveillance Systemdatabase	NR	49.0	All-cause mortality rate	*ICD-9* code	Observation period: 2000-2018; mortality analysis: 2000-2017	Total: 2 170 045; ADHD 1: 80 755; ADHD 2: 182 840; control: 1 906 450	Good
Fitzgerald et al,^30^ 2019	Denmark	Danish nationwide registers	NR	30.79%	Death by suicide	*ICD-8* and *ICD-10*	1995-2014; 164 113 Person-years (ADHD group)	Total: 2 924 780; ADHD: 32 540	Good
Chen et al,^31^ 2022	Taiwan	Taiwan’s National Health Insurance Research Database	NR	21.95	All-cause mortality, natural cause, unnatural cause, suicide, unintentional injury	*ICD-9* code	Index date to December 31, 2013 (variable follow-up)	Total: 1 361 192; ADHD medication: 68 096; no ADHD medication: 68 096	Good
Vasiliadis et al,^32^ 2024	Quebec	The Quebec Integrated Chronic Diseases Surveillance System	NR	28.75	Death by suicide	*ICD-9* and *ICD-10* code	2000-2021; From first ADHD diagnosis or medication claim until age 25 y, death, or study end	Total:217 192; ADHD medication: No ADHD medication:	Good
Li et al,^33^ 2024	Sweden	Swedish national registries	17.4	41.3	All-cause mortality, unnatural cause mortality, natural cause mortality	*ICD-10* code	From ADHD diagnosis until death, emigration, 2 y after diagnosis, or December 31, 2020; 5-y follow-up in sensitivity analysis	Total: 148 578	Good
London et al,^34^ 2022	United States	Data from the 2007 and 2012 National Health Interview SurveySample Adult File linked to National Death Index data through 2015	NR	54.5	All-cause mortality and mortality status	Self-reported ADHD diagnosis status	2007/2012-2015; 9574 Person-years (ADHD group)	Total: 56 433	Good
Barbaresi et al,^35^2012	Minnesota	Birth cohort consisting of all children born between January 1, 1976, and December 31, 1982, to mothers residing in the townships comprising Minnesota Independent School District 535, who continued to live in Rochester until ≥5 y of age and who granted permission for use of their medical records for the study	27 (NR)	56.8	Cause-specific mortality: medically related, suicide, homicide, unintentional injury	Symptoms consistent with *DSM-IV-TR*	From ADHD diagnosis to death, prospective study participation, or last known alive (follow-up through 2009)	Total: 5313; ADHD: 367; control: 4946	Good
Ljung et al,^36^ 2014	Sweden	Multiple Swedish national registers	NR	50.2	Death by suicide	*ICD-9* and *ICD-10* code	1987-2009; Variable follow-up (matched on birth year; up to age 40 y)	Total: 310 242; ADHD:51 707; control: 258 535	Good
Yeh et al,^37^ 2019	United States	Mental Health Research Network, a consortium of 13 health care systems across the US	NR	52.51	Death by suicide	*ICD-0-CM* code	Case-control study; 2000–2013; 1-y exposure assessment period before index date	Total: 267 374; ADHD: 2647; control: 267 400	Good
Vasiliadis et al,^38^ 2025	Quebec	Quebec Integrated Chronic Diseases Surveillance System, which links the health insurance registry, vital statistics death registry, medical physician claims registry, hospitalization registry, and pharmaceutical registry	31.7 (9.3)	28.8	Death by suicide	ICD-10 code	2000-2021; Nested case-control; ≥365 d of continuous drug-insurance coverage before index date	Total: 2832; ADHD: 472; control: 2360	Good
Tuan et al,^39^ 2022	US	Used EHRs data of 57 health care organizations from the TriNetX research network database	35.3 (12.6)	43.44	Mortality due to COVID-19 complications	*ICD-10* code and at least 1 prescription of stimulant medication	30 d After index COVID-19 infection (study period: January 2020-June 2021)	Total: 70 269; ADHD: 28 011; control: 42 258	Good
Rice et al,^40^ 2025	Helsinki	Patient EHRs stored in the Department of Veterans Affairs Corporate Data Warehouse (mortality outcomes identified with the Department of Veterans Affairs and Department of Defense Mortality Data Repository, which links information on veterans with death certificate data from the Centers for Disease Control and Prevention National Death Index)	39.4 (12.4)	17.82	Suicide mortality, overdose, external cause mortality	*ICD-10* code	Mean (SD) follow-up: 29.8 (14.7) mo	Total: 73 177	Good

Abbreviations: ADHD, attention-deficit/hyperactivity disorder; *DSM-IV*, *Diagnostic and Statistical Manual of Mental Disorders *(Fourth Edition); *DSM-IV-TR*, *Diagnostic and Statistical Manual of Mental Disorders* (Fourth Edition, Text Revision); EHR, electronic health record; *ICD-8*, *International Classification of Diseases, Eighth Revision*; *ICD-9*, *International Classification of Diseases, Ninth Revision*; *ICD-9-CM, International Classification of Diseases, Ninth Revision, Clinical Modification*;* ICD-10*, *International Classification of Diseases, Tenth Revision*; NR, not reported.

### Meta-Analyses

Twelve studies^5,23,24,25,26,27,28,29,30,31,32,33^ reported outcomes that could be used in meta-analyses. Five meta-analyses were conducted for the following outcomes: all-cause mortality (n = 9), suicide (n = 5), unintentional or intentional or injury (n = 4), natural-cause mortality (n = 3), and all-cause mortality in individuals with ADHD using ADHD medication (n = 3). The other 7 studies^30,31,32,33,35,36,37^ met inclusion criteria for the current review but were not included in the meta-analyses because (1) they reported noncomparable metrics,^30,31,32,33^ (2) they reported mortality outcomes that could not be grouped with the other studies (COVID-19 mortality),^35^ or (3) the number of studies was insufficient to include in the meta-analysis (eg, suicide as the mortality outcome in people taking ADHD medication).^34,37^ Results of the meta-analysis for all-cause mortality, suicide, unintentional or intentional injury mortality, and natural-cause mortality are depicted in Figure 2, and the results for all-cause mortality for individuals with ADHD using ADHD medications are depicted in Figure 3.

**Figure 2.**
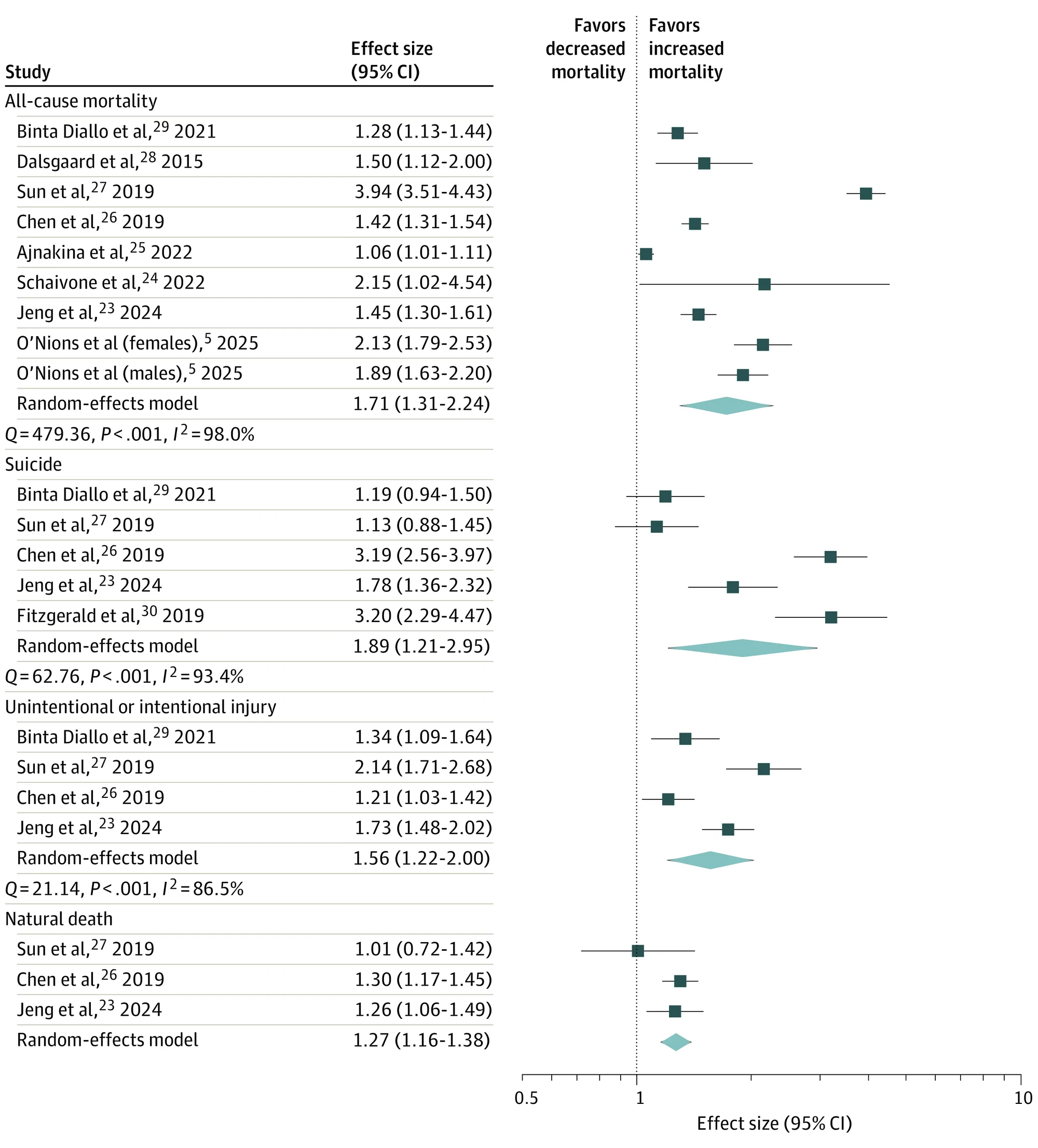
Forest Plot Showing Attention-Deficit/Hyperactivity Disorder Mortality Forest plot for all-cause, suicide, unintentional and intentional injury, and natural death outcomes. Error bars indicate 95% CIs; diamonds, overall effect.

**Figure 3.**
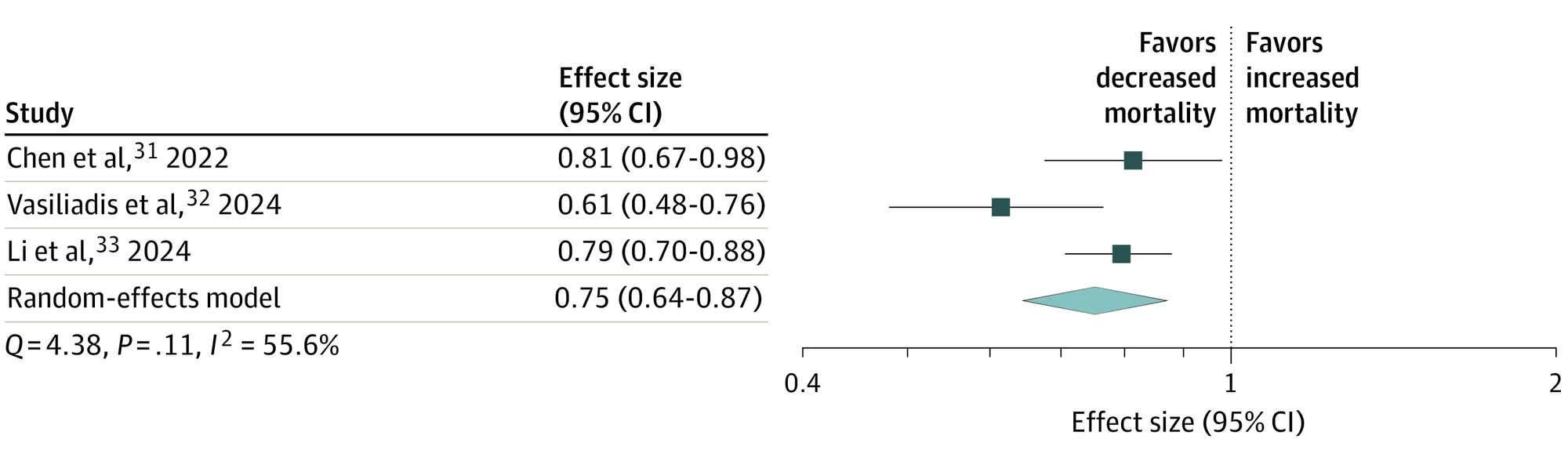
Forest Plot Showing Attention-Deficit/Hyperactivity Disorder (ADHD) Medication Mortality Forest plot for ADHD medication use in people with ADHD and all-cause mortality. Error bars indicate 95% CIs; diamond, overall effect.

The meta-analysis found higher rates of all-cause mortality in individuals with ADHD (hazard ratio [HR], 1.71; 95% CI, 1.31-2.24; *P* < .001). There were also significant levels of heterogeneity in the model (*I*^2^ = 98%; *Q* = 479.36; *P* < .001). There were also significant associations between ADHD and cause-specific mortality, including death by suicide (HR, 1.89; 95% CI, 1.21-2.95; *P* = .005), unintentional or intentional injury (HR, 1.55; 95% CI, 1.22-2.00; *P* < .001), and natural-cause death (HR, 1.27; 95% CI, 1.16-1.38; *P* < .001). There were significant levels of heterogeneity in suicide (*I*^2^ = 93%; *Q* = 62.76; *P* < .001) and death by unintentional or intentional injury (*I*^2^ = 87%; *Q* = 21.14; *P* < .001) meta-analyses but not in the natural-cause model (*I*^2^ < 1%; *Q* = 1.94; *P* = .37).

The meta-analyses testing associations between ADHD medication use in people with ADHD and all-cause mortality showed a significant association between ADHD medication use and lower all-cause mortality (HR, 0.75; 95% CI, 0.64-0.87; *P* < .001). There were moderate levels of heterogeneity (*I*^2^ = 55%), although the Q statistic was not statistically significant (*Q* = 4.38; *P* = .11), potentially reflecting limited power to detect heterogeneity.

The Egger regression test indicated no evidence of publication bias for all-cause mortality (*t* = 0.58; *P* = .56), suicide (*t* = 0.69; *P* = .49), unintentional or intentional injury (*t* = 0.81; *P* = .41), and natural cause (*t* = -1.35; *P* = .18). There was also no evidence of publication bias for all-cause mortality in those taking medication (*t* = –0.96; *P* = .33) (eFigures 3-7 in Supplement 1). The Egger statistics should be treated with caution due to a small number of studies within meta-analyses (<10).

To evaluate the influence of the polygenic risk scores study on heterogeneity, sensitivity analyses were performed with this study excluded. The results of the sensitivity analysis are presented in eFigure 8 in Supplement 1, with the corresponding funnel plot shown in eFigure 9 in Supplement 1.

## Discussion

ADHD was associated with increased mortality across outcomes, including a 1.7-fold higher hazard of all-cause mortality, a 1.9-fold higher hazard of suicide, a 1.5-fold higher hazard of unintentional or intentional injury, and a 1.3-fold increase in natural-cause death, with substantial variation among studies. ADHD medication use was associated with lower all-cause mortality. This finding highlights the importance of ADHD as a long-term public health concern across the life course. There was substantial heterogeneity among studies; however, all studies consistently showed higher mortality in the ADHD groups. This finding suggests that heterogeneity is likely due to differences in the magnitude of the effect rather than disagreement about the direction of the association.

There are multiple potential explanations for the observed association between ADHD and higher mortality rates, which may differ depending on mortality type. There was a significant association between ADHD and death by suicide. This may be observed due to higher rates of co-occurring mental health challenges in people with ADHD, including lifetime history of depression and anxiety disorders,^41^ posttraumatic stress disorder,^42^ schizophrenia,^43^ and substance use disorders.^44^ Given the frequent co-occurrence of ADHD and autism, the observed association between ADHD and mortality may partly reflect autism-related factors and the broader contribution of neurodevelopmental comorbidity. Additionally, people with ADHD are disproportionately more likely to experience societal exclusion, disadvantage, and stigma,^45,46^ which may increase the risk of suicidality. These factors may also intersect with core ADHD traits, such as differences in impulse control^47^ and decision-making,^48^ further increasing risk.

Additionally, a significant association between ADHD and higher rates of death by unintentional or intentional injury was shown. This association may be reflected by multifactorial mechanisms. For example, co-occurring conditions, such as substance use disorders, that are more common in ADHD^49^ may exacerbate risk for unintentional harm. Research shows that ADHD is also associated with an increased risk of involvement in serious transport accidents^50^ and of unintentional injury.^51^ This may be associated with differences in attention, hyperactivity, and impulse control in people with ADHD.^52^

We found a significant association between ADHD and higher rates of natural-cause death. Studies^53,54^ have reported increased risk of a range of physical health conditions in people with ADHD, including cardiovascular disease^7^ and metabolic disorders.^8^ Lifestyle-related factors, such as smoking,^55^ poor diet,^56^ and sleep disorders,^57^ may partly explain these associations. Additionally, challenges with attention and organization may affect the ability to adhere to medical treatment plans as well as engagement with health-promoting practices and health care services, further increasing vulnerability to adverse health conditions and associated mortality in this population. Co-occurring mental health conditions, such as anxiety and depression, may also contribute to this observed association through increased levels of stress and difficulties with self-care. Social determinants, such as socioeconomic disadvantage^58^ and lower educational attainment,^59^ may have an additional weighting effect, potentially contributing to decreased health literacy and higher overall mortality.

Our findings are broadly consistent with the previously published review by Catalá-López et al,^12^ which also found increased all-cause mortality and mortality from unnatural causes among people with ADHD. However, whereas that review^12^ found no clear evidence of increased natural-cause mortality, our analysis identified a modest association with natural-cause mortality. This difference may reflect the inclusion of more recently published studies or differences in the studies and effect estimates included. Our review also extends existing evidence by providing separate estimates for suicide and unintentional or intentional injury mortality and by examining the association between ADHD medication use and mortality. The high heterogeneity in both the current review and the review by Catalá-López et al^12^ indicates that the magnitude of excess mortality varies considerably across populations and study contexts.

Taken together, the current evidence suggests that there are multiple factors that may increase risk for mortality outcomes in people with ADHD, reinforcing the importance of support for managing health risks in this population and attention to addressing structural determinants of poor health disproportionately experienced by people with ADHD. This finding highlights the importance of sustained support and interventions targeting not only symptom management but also broader psychosocial functioning across the lifespan.

This review also showed a significant association between ADHD medication use and reduced rates of all-cause mortality in people with ADHD. This finding reinforces the importance of accessible diagnosis and treatment for ADHD across the lifespan. Previous research highlights that ADHD medication can support people with ADHD in multiple domains of life, including reducing risk of injury,^60^ mental health conditions,^61^ suicidal behavior and mortality^17,23^ and substance use disorders,^62^ in addition to supporting management of core ADHD-related traits.^63^ Reduction of risk across these factors may contribute to the observed reduction in all-cause mortality rates. However, this association should be interpreted cautiously because the medication analysis was based on only 3 studies and may be subject to residual confounding and confounding by indication, given potential differences between treated and untreated individuals in health care access, socioeconomic status, illness severity, treatment adherence,^64^ and psychiatric comorbidity. Therefore, these findings should not be interpreted as evidence of a causal protective effect of ADHD medication on mortality,

### Limitations

This review also has limitations. There were significant levels of heterogeneity reported in some of the models, which suggests potential variation across the included studies. Notably, the included studies were cross-cultural, which may reflect differences in diagnostic criteria, access to, and models of physical and psychological health care and social attitudes toward ADHD, which may influence the outcomes reported. Many of the included studies exhibit gender imbalances, frequently dominated by male participants, which may exclude differences with sex-specific risk, particularly given emerging evidence of differences in presentation and underdiagnosis of ADHD in females. However, it could also be argued that these gender imbalances are reflective of current prevalence statistics.^65^ Differences in sample sizes across the included studies may also contribute because smaller studies typically produce less precise estimates than larger registry-based studies. Changes over time in recognition and management of ADHD and co-occurring conditions may also have contributed to observed heterogeneity. Although larger cohort studies may provide more accurate longitudinal outcomes, they can sometimes rely on less rich clinical data that capture individual differences within populations. Although we used fully adjusted models when extracting effect sizes in this review, there was still variation among studies in which covariates were accounted for, such as socioeconomic status, which may contribute to heterogeneity. The small number of studies, uneven distribution of study characteristics, and inconsistent reporting meant that reliable subgroup analyses or meta-regression were not possible. Sensitivity analysis was conducted excluding the study using ADHD polygenic risk scores. This produced findings consistent with the primary analysis. Pooled estimates should be interpreted as average associations across diverse populations and study contexts rather than as being universally applicable,

Future research should consider psychosocial and sociodemographic factors to understand how being female,^66^ being of a minoritized race or ethnicity, and having experienced childhood maltreatment^67^ may contribute to the risk of mortality in people with ADHD. More diverse and representative samples are needed to improve generalizability and to identify inclusive pathways for intervention. Subgroups of people with ADHD who experience the highest mortality risk should be examined to support informing of initiatives to reduce premature mortality in people with ADHD. Clinically, these findings underscore the importance of long-term, comprehensive care for individuals with ADHD, with particular attention to identifying and addressing modifiable factors that may contribute to excess mortality risk. The association between medication use and lower mortality also highlights the importance of equitable access to evidence-based ADHD assessment and treatment.

## Conclusions

In this systematic review and meta-analysis, findings highlighted associations between ADHD and higher rates of mortality across multiple causes, including all-cause mortality, suicide, unintentional or intentional injury, and natural causes. This study also found that ADHD medication was associated with decreased rates of mortality in ADHD populations. These results reinforce the importance of targeted intervention and access to diagnosis and treatment for ADHD individuals across the lifespan.

## Notes

Supplement 1. **eFigure 1.** Full search terms **eFigure 2.** List of all excluded articles at full text with brief justification **eFigure 3.** Funnel plot for all-cause mortality **eFigure 4.** Funnel plot for suicide mortality **eFigure 5.** Funnel plot for accident or injury deaths **eFigure 6.** Funnel plot for natural cause of death **eFigure 7.** Funnel plot for all-cause mortality in medication use **eFigure 8.** Sensitivity analysis forest plot **eFigure 9.** Sensitivity analysis funnel plot

Supplement 2.
Data Sharing Statement
