# Supplementary material for: Mortality Among People With Attention-Deficit/Hyperactivity Disorder: A Systematic Review and Meta-Analysis
Source: JAMA Netw Open. 2026 Oct 19;9(10):e2639448. doi: 10.1001/jamanetworkopen.2026.39448 (PMC13681224; doi:10.1001/jamanetworkopen.2026.39448)
Supplement: Supplementary file 1 [file jamanetwopen-e2639448-s001.pdf]

Supplemental Online Content

Corrigan L, Heywood L, Stewart GR, et al. Mortality among people with attention-deficit/hyperactivity disorder: a systematic review and meta-analysis. *JAMA Netw Open*. 2026;0(10):e2639448. doi:10.1001/jamanetworkopen.2026.39448

eFigure 1. Full search terms

eFigure 2. List of all excluded articles at full text with brief justification

eFigure 3. Funnel plot for all-cause mortality

eFigure 4. Funnel plot for suicide mortality

eFigure 5. Funnel plot for accident or injury deaths

eFigure 6. Funnel plot for natural cause of death

eFigure 7. Funnel plot for all-cause mortality in medication use

eFigure 8. Sensitivity analysis forest plot

eFigure 9. Sensitivity analysis funnel plot

This supplemental material has been provided by the authors to give readers additional information about their work.

© 2026 Corrigan L et al. *JAMA Network Open*.

eFigure 1. Full search terms

The full list of search terms applied to each database listed.	(ADHD OR "Attention Deficit Hyperactivity Disorder" OR "Attention Deficit Disorder with Hyperactivity" OR hyperkine* OR HKD OR inattention OR hyperactivity OR impulsivity OR "Attention Deficit/Hyperactivity Disorder" OR "Attention Deficit Disorder") AND (mortalit* OR death OR fatal* OR "life expectancy" OR "years of life lost" or suicide*).

© 2026 Corrigan L et al. *JAMA Network Open*.

eFigure 2: List of all excluded articles at full text with brief justification

Title	Author	Year	Justification
1. Suicidal behavior in patients with severe mental disorders prior to and during the COVID-19 pandemic.	Mittendorfer-Rutz et al	2024	This study investigates severe mental disorders therefore ADHD is not main predictor for mortality and cannot be included in the meta-analysis.
2. Associations of asthma and psychiatric comorbidities on suicide mortality among community adolescents: A 20-year cohort study.	Chen et al	2024	This study focusses on psychiatric comorbidity as a confounder between asthma and suicide mortality. ADHD is therefore not investigated as primary predictor for mortality therefore cannot be included in the meta-analysis.
3. Cause-specific mortality and comorbid neurodevelopmental disorder in 167,515 patients with bipolar disorder: An entire population longitudinal study.	Cho et al	2024	This study was excluded because it investigated mortality among individuals with bipolar disorder and a comorbid neurodevelopmental disorder. As ADHD was examined in the context of comorbid bipolar disorder, the mortality rates cannot be attributed specifically to ADHD. The presence of bipolar disorder may independently influence mortality risk and therefore confound the association between ADHD and mortality. Including this study in

© 2026 Corrigan L et al. *JAMA Network Open*.

			the meta-analysis could consequently result in an inaccurate estimate of mortality associated with ADHD.
4. Comorbidity of anxiety/affective, conduct/antisocial, attention deficit, and alcohol use disorders with suicidal behaviors in an American Indian community sample of adults and adolescents.	Ehlers et al	2023	ADHD was examined alongside a range of psychiatric conditions rather than being the specific exposure of interest and The outcome was suicidal thoughts/suicidal acts, with suicide deaths included within the suicidal-acts category, rather than mortality being analysed as a distinct outcome.
5. Comparative Effectiveness of Pharmacotherapies for the Risk of Attempted or Completed Suicide Among Persons With Borderline Personality Disorder.	Lieslehto et al	2023	ADHD not the primary exposure of interest. The study focused on pharmacological treatment among individuals with borderline personality disorder (BPD), comparing multiple medication classes in relation to suicidal outcomes. ADHD medication was examined as one of several pharmacotherapies rather than ADHD being investigated as the primary predictor of mortality
6. Effect of comorbid ADHD on mortality in women with borderline personality disorder.	Kouppis et al	2022	This study was excluded because it investigated mortality among individuals with borderline personality disorder and co-

© 2026 Corrigan L et al. *JAMA Network Open*.

			occurring ADHD. As ADHD was examined in the context of borderline personality disorder (BPD), the mortality rates cannot be attributed specifically to ADHD. The presence of BPD may independently influence mortality risk and therefore confound the association between ADHD and mortality. Including this study in the meta-analysis could consequently result in an inaccurate estimate of mortality associated with ADHD.
7. Risk and mortality of testicular cancer in patients with neurodevelopmental or other psychiatric disorders.	Jansson	2023	Predictor is neurodevelopmental disorder with ADHD included but ADHD is not an independent measure as a predictor of mortality.
8. Mortality in individuals with disruptive behavior disorders diagnosed by specialist services - A nationwide cohort study.	Scott et al	2017	ADHD not the primary exposure of interest. This study focussed on the association between disruptive behaviour disorders and mortality and ADHD was examined as a comorbid condition and as a potential mediator of mortality risk within disruptive behaviour disorder populations.

© 2026 Corrigan L et al. *JAMA Network Open*.

9. Factors related to childhood suicides: analysis of the Queensland Child Death Register.	Soole et al	2014	ADHD not the primary exposure/predictor of interest. The study investigated demographic, psychosocial and psychiatric factors associated with suicide among children and adolescents, with ADHD considered as one of several psychiatric factors rather than as the primary predictor of mortality.
10. Long term safety of ADHD medication in patients with schizophrenia spectrum disorders.	Luykx et al	2025	This study investigates the long term safety of ADHD medications in patients with schizophrenia spectrum disorders. As the ADHD population in this study is in the context of schizophrenia spectrum disorder populations, mortality in ADHD alone cannot be inferred.
11. Socioeconomic and gender inequalities in mental disorders among adolescents and young adults.	Mar et al	2024	The study investigated socioeconomic and gender inequalities in mental disorders and mortality, with ADHD assessed as one of several mental health diagnoses. Mortality was analysed in relation to socioeconomic status and the presence of a mental health diagnosis, rather than specifically

© 2026 Corrigan L et al. *JAMA Network Open*.

			examining ADHD as a predictor of mortality.
12. Overall and Cervical Cancer Survival in Patients With and Without Mental Disorders.	Herweijer et al	2023	ADHD not the primary exposure/predictor. The study investigated the association between pre-existing mental disorders collectively and overall/cervical cancer-specific mortality among patients with cervical cancer. Although ADHD was included as one of the mental disorder diagnoses, mortality was not analysed specifically according to ADHD diagnosis.
13. Post-injury outcomes of children with behavioral health disorders.	Traynor et al	2023	The study investigated the association between behavioural health disorders collectively and in-hospital mortality following traumatic injury. Although ADHD was the most common behavioural health disorder in the cohort, it was included as one of several disorders within the broader BHD exposure and a specific ADHD mortality association was not reported.
14. Outcomes in Trauma Patients With Behavioral Health Disorders.	Harfouche et al	2019	ADHD is categorised under behavioural health disorder but mortality is measured under this

© 2026 Corrigan L et al. *JAMA Network Open*.

			collective term rather than ADHD independently.
15. Prevalence of behavioral health disorders and associated chronic disease burden in a commercially insured health system: findings of a case-control study.	Young et al	2015	Ineligible exposure/outcome analysis. Although ADHD was one of the behavioural health disorders examined, the study focused on the prevalence of behavioural health disorders and associated chronic disease burden. The reported 10-year mortality risk was derived from the Charlson Comorbidity Index rather than observed mortality, and no specific association between ADHD and mortality was assessed.
16. Excess Mortality from Mental, Neurological, and Substance Use Disorders in the Global Burden of Disease Study 20	Charlson et al	2016	no ADHD-specific mortality estimate. ADHD was included within the broader category of childhood/disruptive behavioural disorders alongside conduct disorder; however, the study did not estimate cause-specific or excess mortality for this category. Therefore, no association between ADHD and mortality was reported.

© 2026 Corrigan L et al. *JAMA Network Open*.

17. The hidden risk factors behind of suicidal behavior in medical students: a cross-sectional cohort study in Mexico.	Martinez-Fierro et al	2025	In this study, suicidal behaviour was defined as ideation and attempts. Death by suicide was not an outcome, therefore mortality rates could not be measured in this study and is therefore excluded.
18. Genetic Confounding in the Association Between Traumatic Brain Injury and Mental Disorder or Suicide.	Ostergard et al	2025	The primary outcome in this study was traumatic brain injury and secondary outcomes were polygenic risk scores for other mental disorders including ADHD. Due to the primary outcome not being mortality or death, this paper was excluded.
19. Phenome-wide diagnostic comparison among suicide deaths and living individuals with chronic pain diagnoses.	DiBlasi et al	2024	This study population was people with a chronic pain diagnosis. As ADHD was not the primary population of the sample, this study was excluded from the review.
20. ADHD Pharmacotherapy and Mortality in Individuals With ADHD.	Li et al	2024	This paper is a commentary and is not an original piece of research presenting new findings and was therefore excluded.
21. The shared genetic architecture of suicidal behaviour and psychiatric disorders: A	Kootbodien et al	2023	This study defines suicidal behaviour as suicidal ideation, self-harm, suicide attempt and completed suicide. The study did not examine the association

© 2026 Corrigan L et al. *JAMA Network Open*.

genomic structural equation modelling study.			between ADHD and completed suicide as an outcome. The completed-suicide phenotype was analysed separately, in an East Asian population, and was not included in the genomic structural equation model examining the shared genetic architecture of ADHD and suicidal behaviour. The ADHD-related analyses were based on non-fatal suicidal behaviour phenotypes, including suicide attempt, self-harm and suicidal ideation.
22. Attention-Deficit/Hyperactivity Disorder and Suicidal Risk in Major Depression: Analysis of 141,530 Adolescent Hospitalizations.	Zahid et al	2020	Excluded because the study assessed suicidal behaviours among adolescents hospitalised with major depressive disorder, rather than completed suicide/suicide mortality. The study used cross-sectional inpatient data and did not ascertain deaths from suicide
23. Suicide Risk Among Adolescents With ADHD: An Overview From the National Inpatient Sample Data Set.	Trivedi et al	2023	Study examined suicidal ideation and suicide attempts among adolescents with ADHD using National Inpatient Sample hospitalisation data; it did not assess completed suicide/suicide mortality.

© 2026 Corrigan L et al. *JAMA Network Open*.

24. Clinical, psychological, social, and family characterization of suicidal behavior in Chilean adolescents: a multiple correspondence analysis.	Mendez-Bustos et al	2022	Full text not available in English
25. The trend analysis of neurological disorders as major causes of death and disability according to human development, 1990-2019.	Borumandnia et al	2022	Study examined global trends in the incidence of ADHD and other neurological disorders using Global Burden of Disease data; it did not report ADHD-specific mortality or deaths
26. Childhood neurodevelopmental markers and risk of premature mortality: Follow-up to age 60-65 years in the Aberdeen Children of the 1950s study.	Warrilow et al	2021	exposure not specific to ADHD: Study examined associations between childhood neurodevelopmental/behavioural markers and all-cause premature mortality. Although some Rutter B items represented ADHD-related characteristics (e.g., fidgetiness and attention problems), the study did not assess diagnosed ADHD or provide an ADHD-specific mortality estimate.
27. Self-Reported Depressive Symptoms and Suicidality in Adolescents with Attention-Deficit/Hyperactivity Disorder: Roles of Bullying Involvement, Frustration Intolerance, and Hostility.	Liu et al	2021	Study assessed suicidal ideation or suicide attempt in adolescents with ADHD; it did not assess completed suicide or suicide mortality.

© 2026 Corrigan L et al. *JAMA Network Open*.

28. Risk factors for pre-adolescent onset suicidal behavior in a high-risk sample of youth.	Shen et al	2021	Study examined suicidal behaviour, including suicide attempts and severe suicidal ideation, rather than completed suicide/suicide mortality.
29. Developmental profiles of childhood attention-deficit/hyperactivity disorder and irritability: association with adolescent mental health, functional impairment, and suicidal outcomes.	Galera et al	2021	Study examined adolescent suicidal behaviours (suicidal ideation/suicide attempt) associated with childhood ADHD/irritability trajectories; it did not assess completed suicide or suicide mortality.
30. Risk Factors of Suicide Death Based on Psychological Autopsy Method; a Case-Control Study.	Rasouli et al	2019	exposure not specific to ADHD: Study examined risk factors for completed suicide using psychological autopsy. Although suicide death was the outcome, ADHD was not specifically assessed or reported as an exposure, and no ADHD-specific association with suicide death was provided.
31. Adult ADHD and suicide.	Furczyk et al	2014	Ineligible study design: Not an original mortality study: Review article discussing the association between adult ADHD and suicidal behaviour; did not provide original data on ADHD and completed suicide mortality.

© 2026 Corrigan L et al. *JAMA Network Open*.

32. Legg-Calve-Perthes disease and the risk of ADHD, depression, and mortality.	Hailer et al	2014	Study examined mortality (including completed suicide) in individuals with Legg-Calvé-Perthes disease, with ADHD assessed as an outcome. It did not examine mortality risk among individuals with ADHD or provide an ADHD–mortality association
33. Comparative mortality risk in adult patients with schizophrenia, depression, bipolar disorder, anxiety disorders, and attention-deficit/hyperactivity disorder participating in psychopharmacology clinical trials.	Khan et al	2013	Study examined mortality among participants in psychopharmacology clinical trials across psychiatric disorders, but did not assess the association between ADHD medication use and mortality among people with ADHD.
34. Loss Due to Death and its Association with Mental Disorders in Juvenile Detainees.	Harnisher et al	2015	Study examined bereavement/loss due to death and its association with mental disorders, including ADHD/behavioural disorders. It did not examine mortality among people with ADHD or the association between ADHD medication and mortality.
35. Completed suicide, ideation and attempt in attention deficit hyperactivity disorder.	Impey et al	2012	Ineligible publication type: systematic review

© 2026 Corrigan L et al. *JAMA Network Open*.

36. Psychological variables potentially implicated in opioid-related mortality as observed in clinical practice.	Passik et al	2011	Clinical review/discussion based on clinical experience; no original study of ADHD medication exposure and mortality.
37. Childhood predictors of completed and severe suicide attempts: findings from the Finnish 1981 Birth Cohort Study.	Sourander et al	2009	This study does not use an ADHD group as the population of interest, instead used information about psychopathological conditions more broadly.
38. Stimulants and sudden death: what is a physician to do?.	Wilens et al	2006	This is a commentary article which does not present any original research findings and is therefore excluded.
39. [Medication for ADHD and the risk of cardiovascular mortality].	Langendijk et al	2006	This is a commentary article which does not present any original research findings and is therefore excluded.
40. Arteriosclerotic and other degenerative heart diseases in Finland. I. A death certificate study of the frequency of degenerative heart diseases among males and females	Penttila et al	1975	not ADHD (ADHD was an abbreviation for arteriosclerotic/degenerative heart disease).
41. Can Increased Recovery Rates from Coronavirus be explained by Prevalence of ADHD? An Analysis at the US Statewide Level	Arbel et al	2021	Ecological/state-level study examining associations between population prevalence of ADHD and COVID-19 mortality; did not assess individual-level mortality among people with ADHD.

© 2026 Corrigan L et al. *JAMA Network Open*.

42. Midlife diseases of despair and cardiometabolic risk: testing shared origins in adolescent psychopathology	Dent et al	2024	Study assessed midlife morbidity (including suicidal ideation/attempt and cardiometabolic risk), not mortality.
43. Life expectancy is lower in adults with ADHD.	Roush	2025	Non-original commentary/summary of research; no original mortality data.
44. Attention-deficit/hyperactivity disorder and cardiometabolic risk profile in patients with acute myocardial infarction: data from the SWEDEHEART registry.	Yao et al	2025	Mortality was assessed only among patients with acute myocardial infarction, rather than the ADHD population generally.
45. Association between methylphenidate use and long-term cardiovascular risk in paediatric patients with attention deficit and hyperactivity disorder.	Liao et al	2024	This study focussed on cardiovascular risk with the outcome being major cardiovascular event, mortality was included as a component of a broader cardiovascular safety analysis rather than being the primary outcome of interest.
46. Comorbid health conditions in people with attention-deficit/hyperactivity disorders: An umbrella review of systematic reviews and meta-analyses.	Kang et al	2024	Ineligible study design (review)

© 2026 Corrigan L et al. *JAMA Network Open*.

47. ADHD Pharmacotherapy and Mortality-Reply.	Li et al	2024	Ineligible publication type - reply
48. ADHD Pharmacotherapy and Mortality.	Zarkowski	2024	Ineligible publication type – letter to the editor
49. ADHD Pharmacotherapy and Mortality.	Richards-Belle et al	2024	Ineligible publication type – letter to the editor
50. ADHD genetic burden associates with older epigenetic age: mediating roles of education, behavioral and sociodemographic factors among older adults.	Arperwong et al	2023	The paper is an original research article, and it does examine an ADHD-related exposure: an ADHD polygenic score (ADHD-PGS). However, the actual outcome is GrimAge, an epigenetic measure of biological/accelerated ageing. It is not observed mortality, deaths, or a mortality rate.
51. Higher Risk of Mortality for Individuals Diagnosed With Autism Spectrum Disorder or Attention-Deficit/Hyperactivity Disorder Demands a Public Health Prevention Strategy.	Barkley et al	2022	Ineligible study design - Opinion article discussing findings from a systematic review/meta-analysis; did not provide original mortality data.
52. [ADHD in adults - history, epidemiology, and neuroscience].	Borg Skoglund et al	2022	Full text not available in English
53. Cardiovascular Effects of Drugs Used to Treat Attention-Deficit/Hyperactivity Disorder Part 2: Impact on Cardiovascular Events and	Fay et al	2019	Ineligible study design - narrative review of cardiovascular effects of ADHD medications; no original ADHD–mortality analysis.

© 2026 Corrigan L et al. *JAMA Network Open*.

Recommendations for Evaluation and Monitoring.			
54. Considerations in the Care of Athletes With Attention Deficit Hyperactivity Disorder.	Pujalte et al	2019	Ineligible study design - This is explicitly a “General Review” that synthesises existing literature on ADHD in athletes. It does not provide original data examining mortality among people with ADHD.
55. Attention deficit hyperactivity disorder.	Thapar et al	2016	Ineligible study design - This is a review article, not an original mortality study
56. Long-QT Syndrome and Therapy for Attention Deficit/Hyperactivity Disorder.	Zhang et al	2015	The population consists of people with long-QT syndrome, not a general ADHD population, and ADHD medication is the exposure. Deaths were recorded but were too few to permit a treatment-related mortality analysis; the primary outcome was broader cardiac events.
57. Adverse drug reactions - examples of detection of rare events using databases.	Chan et al	2015	Review article summarising existing studies of rare adverse events; no original analysis of mortality in people with ADHD.
58. Effect of drugs on the risk of injuries in children with attention deficit hyperactivity	Dalsgaard et al	2015	Ineligible outcome -study examined injuries and emergency ward visits rather than mortality;

© 2026 Corrigan L et al. *JAMA Network Open*.

disorder: a prospective cohort study.			deaths were reported descriptively but mortality was not analysed as an outcome
59. Attention deficit hyperactivity disorder and premature death.	Farone	2015	Ineligible publication type - editorial/commentary discussing findings from an original ADHD mortality cohort study; no original mortality data.
60. Injury prevention by medication among children with attention-deficit/hyperactivity disorder: a case-only study.	Mikolakczyk et al	2015	Ineligible outcome - study examined hospitalisations for injury and brain injury rather than mortality
61. Prevalence and correlates of attention-deficit hyperactivity disorder symptoms in Korean college students.	Kwak et al	2015	Ineligible outcome - study examined ADHD symptoms in relation to depression, alcohol use, obesity and lifetime suicidal behaviour, rather than mortality.
62. ADHD is a risk factor for premature death, Danish study shows.	Young	2015	Ineligible publication type - BMJ news article reporting findings from the Danish ADHD mortality cohort study; does not present original mortality data.
63. Serious transport accidents in adults with attention-deficit/hyperactivity disorder and the effect of medication: a population-based study.	Chang et al	2014	Ineligible outcome - Study assessed serious transport accidents, defined as hospitalisation or death due to transport trauma, rather than mortality as a standalone outcome.

© 2026 Corrigan L et al. *JAMA Network Open*.

64. Does childhood attention-deficit/hyperactivity disorder predict risk-taking and medical illnesses in adulthood?	Ramos-Olazagasti et al	2013	Ineligible outcome - although mortality was reported, the medication analysis assessed psychostimulant exposure in relation to cardiovascular disease rather than mortality.
65. Completed suicide, ideation and attempts in attention deficit hyperactivity disorder.	James	2012	Ineligible publication type - editorial commentary discussing a systematic review of ADHD and suicidality - no original mortality data.
66. How to predict the impact of methylphenidate on cardiovascular risk in children with attention deficit disorder: methylphenidate improves autonomic dysfunction in children with ADHD.	Buchlorn	2012	Ineligible outcome - study assessed heart-rate variability/autonomic dysfunction as a cardiovascular risk marker, not observed mortality.
67. Cardiovascular safety of ADHD medications: rationale for and design of an investigator-initiated observational study.	Hennessy et al	2010	Ineligible study design - study protocol/design paper describing a planned observational study; no original mortality results reported.
68. Stimulant treatment of ADHD and risk of sudden death in children.	Vitiello et al	2009	Ineligible publication type - editorial discussing an original case-control study of stimulant use and sudden death; no original mortality data.
69. Attention-deficit hyperactivity disorder (ADHD) treatment and sudden death.	Besag	2009	Ineligible publication type - commentary discussing an original cohort study of ADHD

© 2026 Corrigan L et al. *JAMA Network Open*.

			drug treatment and mortality; no original mortality data.
70. Stimulant-drug therapy for attention-deficit disorder (with or without hyperactivity) and sudden cardiac death.	Knight	2007	Ineligible publication type - commentary discussing stimulant therapy and sudden cardiac death; no original ADHD mortality data.
71. ADHD Treatment and Mortality Risk		2024	Ineligible publication type – no original mortality data.
72. ADHD Pharmacotherapy and Mortality-Reply	Li et al	2024	Ineligible publication type – reply – no original mortality data
73. Comorbidity, trauma, and mortality in children and young adults using ADHD medications in Quebec between 2000 and 2020	Lunghi et al	2022	Ineligible publication type - conference abstract; does not provide a full original research report with sufficient mortality data for extraction.
74. ADHD Medication Use and Psychiatric Comorbidity, Trauma and Mortality in Children and Young Adults: A Cohort Study from Quebec, Canada	Lunghi et al	2022	Irretrievable and Ineligible publication type - conference abstract; does not provide a full original research report with sufficient mortality data for extraction.
75. Attention deficit hyperactivity disorder associated with increased mortality	Mellis	2015	Ineligible publication type - brief commentary on the association between ADHD and mortality; no original mortality data.

© 2026 Corrigan L et al. *JAMA Network Open*.

76. HEALTH OUTCOMES AND RISK FOR MORTALITY IN OLDER ADULTS WITH ATTENTION DEFICIT/HYPERACTIVITY DISORDER SYMPTOMS	Cooper et al	2024	Irretrievable
77. Potentially fatal cardiomyopathy: An unexpected consequence of therapy for Attention Deficit Hyperactivity Disorder with lisdexamfetamine	Pollak	2021	Ineligible publication type - It is a case report/case description, rather than an epidemiological study evaluating mortality risk or the association between ADHD medication and mortality in an ADHD population.
78. A Randomized Thorough QT Trial Using Concentration-QT Analysis to Evaluate the Effects of Centanafadine on Cardiac Repolarization	Turkoglu et al	2025	Ineligible outcome - Although centanafadine is an ADHD medication, participants were healthy adults rather than people with ADHD, and the study assessed QTc interval/cardiac repolarisation, not medication-associated mortality. No deaths occurred during the study.
79. ADHD medications may be linked to sudden unexplained death		2009	Irretrievable
80. No increased risk of heart attack, stroke or sudden cardiac death in adults with ADHD-Medication		2012	Irretrievable

© 2026 Corrigan L et al. *JAMA Network Open*.

81. Trouble déficit de l'attention/hyperactivité (TDA/H) et conduites à risques chez l'enfant et l'adolescent: Cas particulier des accidents et des blessures non intentionnelles = Attention deficit disorder hyperactivity (ADHD) and risky behavior in child and adolescent: Example of unintentional acci…	Michel et al	2017	Full text not available in English
82. Men with ADHD in compulsory care for substance abuse: Characterization and treatment	Muld et al	2022	Ineligible publication type - doctoral thesis; mortality data were reported within a thesis study comparing pharmacologically treated and untreated adults with ADHD/SUD.
83. Attention-deficit/hyperactivity disorder and risk of suicide/self-harm…Jeng JS, Huang HH, Chang WH, et al. Longitudinal study on all-cause and suicide mortality among individuals with attention deficit hyperactivity disorder. European Child & Adolescent Psychiatry; (New York, New York) 2025; v….	Kawanda et al	2025	Ineligible publication type - Letter to the Editor; does not present original mortality data.
84. ADHD Adversely Impacts Health, Mortality Risk, and	Barkley	2020	Ineligible publication type - review/commentary article

© 2026 Corrigan L et al. *JAMA Network Open*.

Estimated Life Expectancy by Adulthood.			summarising existing evidence on ADHD, mortality risk and life expectancy; no original mortality analysis.
85. High Time Preference Is a Key Cognitive Deficit in ADHD: Impact on Daily Life, Impairments, and Life Expectancy.	Barkley	2022	Ineligible publication type - non-original review/commentary article discussing life expectancy in ADHD; no original mortality data.
86. Cohort study finds medications for ADHD reduce mortality risk.	Anonymous	2024	Ineligible publication type - secondary report summarising an original cohort study of ADHD medication and mortality; no original mortality analysis.
87. Groundbreaking study examines ADHD, life expectancy in children and adults.	Canady et al	2019	Ineligible publication type - secondary news report summarising an original study of estimated life expectancy in ADHD; does not present original mortality data.
88. ADHD Pharmacotherapy and Mortality—Reply.	Li et al	2024	Ineligible publication type – reply, does not report any original mortality data.
89. Medication for ADHD and accidental injury	Newcorn et al	2015	Ineligible publication type - commentary discussing an original cohort study of ADHD medication and accidental injuries; does not present original mortality or medication–mortality data.

© 2026 Corrigan L et al. *JAMA Network Open*.

90. In children with ADHD, does use of stimulant medications increase the risk of sudden cardiac death?	Martinez et al	2021	Ineligible publication type - evidence-based commentary summarising research on stimulant use and sudden cardiac death; no original mortality data.
91. Suicidality in Children with ADHD, Grown Up.	Barkley et al	2005	Ineligible outcome - study assessed suicidality/suicidal behaviour in young adulthood rather than mortality or completed death.
92. Evidence-based medicine. Possible association between stimulants used for ADHD and sudden death in children.	Anonymous	2009	Ineligible publication type - evidence-based summary of research on stimulant use and sudden death; no original mortality data.
93. ADHD drugs linked to sudden death.	Anonymous	2009	Ineligible publication type - news report summarising an original case-control study of stimulant use and sudden death; no original mortality data.
94. Keep your eye on…	Anonymous	2022	Ineligible publication type - brief news/summary article reporting on ADHD-related research; no original mortality data.
95. Numeric Error in Figure 1	Anonymous	2022	Ineligible publication type - published erratum correcting a numerical error in a previously published study; not an original research article.

© 2026 Corrigan L et al. *JAMA Network Open*.

96. Risk of cardiac events associated with stimulants in children with ADHD.	Anonymous	2008	Irretrievable
97. Drug update. ADHD drugs linked to cardiac deaths.	Mullett	2009	Irretrievable
98. Chiropractic instead of drugs for ADHD: study links stimulant use to sudden death; media and current research suggest chiropractic is a safe alternative.	Crownfield	2009	Ineligible publication type - secondary media/commentary article reporting findings from an original study of stimulant use and sudden death; no original mortality data.
99. ADHD Pharmacotherapy and Mortality.	Zarkowski	2024	Ineligible publication type - Letter to the Editor commenting on an original ADHD pharmacotherapy–mortality study; does not present original mortality data.
100. Attention-deficit/hyperactivity disorder and adverse health outcomes in adults	Spencer et al	2014	Ineligible outcome - study assessed cardiovascular and metabolic health risk indicators associated with morbidity and mortality, rather than observed mortality.
101. Attention deficit hyperactivity disorder.	Thapar et al	2016	Ineligible publication type - review/seminar providing an overview of ADHD and summarising evidence on premature mortality; no original mortality analysis.

© 2026 Corrigan L et al. *JAMA Network Open*.

102. Study examines heart risk of stimulants in children with ADHD	Anonymous	2008	Ineligible publication type - university news release reporting findings from an original cohort study of stimulant use and cardiovascular outcomes; does not present original mortality data.
103. Adolescents with Elevated Psychopathic Traits are Associated with an Increased Risk for Premature Mortality.	Maurer	2025	ADHD is not main predictor - study primarily examined adolescent psychopathic traits as the predictor of premature mortality; ADHD was assessed only as a supplementary externalising psychopathology measure.
104. Excess mortality and suicide risk in treatment-seeking individuals with schizotypal disorder: A population-based study using Swedish national registers.	Berge et al	2024	ADHD is not main predictor - ADHD was assessed as a comorbidity/predictor within a cohort of individuals with schizotypal disorder, rather than ADHD being the primary population/exposure of interest.
105. Patterns of filled prescriptions and the association with risk of drug-induced death. A population-based nested case-control register study.	Amundsen et al	2024	ADHD is not main predictor - ADHD medication was included as one component of prescription-pattern clusters among individuals with drug-induced death and population controls.

© 2026 Corrigan L et al. *JAMA Network Open*.

106. Mapping the genetic architecture of suicide attempt and suicide death using polygenic risk scores for clinically-related psychiatric disorders and traits.	Otsuka	2023	ADHD is not main predictor - study assessed genetic liability to ADHD using polygenic risk scores rather than ADHD diagnosis as the exposure; therefore it did not assess mortality risk in individuals with ADHD.
107. Association of Pharmacological Treatments and Hospitalization and Death in Individuals With Amphetamine Use Disorders in a Swedish Nationwide Cohort of 13965 Patients.	Hartikainen et al	2023	Ineligible population - study included individuals with amphetamine/methamphetamine use disorder rather than an ADHD-defined population; ADHD was assessed as a comorbidity within the MAUD cohort.
108. Genetic differences between suicide deaths and deaths of undetermined intent.	Edwards et al	2023	ADHD not main predictor - ADHD was included as one of several family genetic risk scores in an analysis comparing suicide deaths with deaths of undetermined intent; the study did not assess mortality risk associated with ADHD.
109. Healthcare utilization and comorbidity shortly before suicide mortality in patients with attention-deficit/hyperactivity disorder: a nested case-control study.	Lee et al	2023	ADHD is not main predictor - study was restricted to individuals with ADHD and compared suicide decedents with living ADHD controls; it did not assess mortality risk associated with ADHD compared with a non-ADHD population.

© 2026 Corrigan L et al. *JAMA Network Open*.

110. General practice database on mortality in adults on methylphenidate: cohort study.	Stricker et al	2022	Ineligible population - cohort was defined by methylphenidate prescribing rather than ADHD diagnosis; an ADHD-defined population was not studied
111. Is There a Relationship Between ADHD and COVID-19 Prevalence and Mortality Indices? An Analysis of Data From 156 Countries.	Rajkumar et al	2022	Insufficient statistical information
112. ADHD, financial distress, and suicide in adulthood: A population study.	Beauchaine et al	2020	Insufficient statistical information
113. ADHD risk genes involved in dopamine signaling and metabolism are associated with reduced estimated life expectancy at young adult follow-up in hyperactive and control children.	Barkley et al	2019	ADHD genetics only - study examined ADHD-related genetic polymorphisms and estimated life expectancy rather than mortality risk associated with ADHD diagnosis.
114. Hyperactive Child Syndrome and Estimated Life Expectancy at Young Adult Follow-Up: The Role of ADHD Persistence and Other Potential Predictors.	Barkley et al	2019	Ineligible outcome - study assessed estimated life expectancy using a life-expectancy calculator rather than observed mortality/death.
115. Postdischarge Suicide and Death in South Korean Children and Adolescents	Paik et al	2018	Ineligible population - ADHD was one diagnostic subgroup within a psychiatric-hospitalization

© 2026 Corrigan L et al. *JAMA Network Open*.

Hospitalized for a Psychiatric Illness.			cohort, not the defined study population.
116. Youth Suicide Deaths: Investigation of Clinical Predictors in a Statewide Sample.	Keeshin et al	2018	Ineligible outcome - study examined clinical characteristics among youth who had already died by suicide; it did not assess mortality risk associated with ADHD.
117. Attention-Deficit/Hyperactivity Disorder and Fatal Accidents in Aviation Medicine.	Laukkala et al	2017	Ineligible study design - descriptive review of fatal aviation accidents involving individuals with ADHD; no comparative analysis of mortality risk associated with ADHD.
118. Attention Deficit Hyperactivity Disorder and adult mortality.	London and Landes	2016	duplicate
119. Drug treatment for attention-deficit/hyperactivity disorder and suicidal behaviour: register based study.	Chen et al	2014	Ineligible outcome - although completed suicide is included, it is combined with suicide attempts in the primary outcome, so it doesn't provide the standalone mortality estimate needed for this meta-analysis.
120. Cardiovascular events and death in children exposed and unexposed to ADHD agents.	Schelleman et al	2011	Ineligible population - participants were identified by ADHD medication prescription rather than confirmed ADHD diagnosis; the study population

© 2026 Corrigan L et al. *JAMA Network Open*.

			was not restricted to individuals with ADHD
121. Mortality associated with attention-deficit hyperactivity disorder (ADHD) drug treatment: a retrospective cohort study of children, adolescents and young adults using the general practice research database.	McCarthy et al	2009	Ineligible population - study examined mortality among individuals receiving ADHD drug treatment rather than mortality associated with ADHD diagnosis compared with a non-ADHD population.
122. Surveillance of prescription drug-related mortality using death certificate data.	Wygowski	2007	Ineligible population - study examined prescription drug-related deaths in the general US population; it did not assess mortality among individuals with ADHD.
123. THE EFFECT OF HIGH POLYGENIC PROPENSITY FOR ADHD AND SOCIOECONOMIC FACTORS ON INCREASING TIMING TO PREMATURE MORTALITY: EVIDENCE FROM ENGLISH LONGITUDINAL STUDY OF AGEING	Fan et al	2022	Ineligible publication type - conference abstract; no full primary research article.
124. Amphetamines, Atomoxetine and the Risk of Serious Cardiovascular Events in Adults	Schelleman et al	2013	Ineligible population - study population was defined by ADHD medication initiation rather than confirmed ADHD diagnosis/identification; it did not

© 2026 Corrigan L et al. *JAMA Network Open*.

			assess mortality associated with ADHD itself.
125. Incidence and trend of cardiac events among children and young adults exposed to psychopharmacological treatment (2006-2018): A nationwide register-based study.	Elmowafi et al	2025	Insufficient statistical information
126. Global burden of ADHD medication-associated cardiovascular disease, 1967-2023: A comparative analysis using the WHO pharmacovigilance database.	Cho et al	2024	Ineligible outcome - study assessed cardiovascular adverse events associated with ADHD medications rather than mortality associated with ADHD diagnosis.
127. Attention-Deficit/Hyperactivity Disorder Medications and Work Disability and Mental Health Outcomes.	Taipale et al	2024	Ineligible outcome - study examined hospitalization, work disability and suicidal behaviour rather than all-cause mortality associated with ADHD
128. Shared genetic architecture between attention-deficit/hyperactivity disorder and lifespan.	Vilar-ribo	2023	Too biologically grounded - study investigated the genetic architecture and genetic liability underlying ADHD and lifespan using GWAS/Mendelian randomisation rather than the association between clinically ADHD and observed mortality.

© 2026 Corrigan L et al. *JAMA Network Open*.

129. The Use of Methylphenidate for Cognitive Enhancement in Young Healthy Adults: The Clinical and Ethical Debates.	Koren et al	2021	Ineligible outcome - article focused on cognitive enhancement and clinical/ethical issues surrounding methylphenidate use; mortality was not assessed as an original study outcome.
130. ADHD medications and cardiovascular adverse events in children and adolescents: cross-national comparison of risk communication in drug labeling.	Sieluk et al	2017	Insufficient statistical information
131. Functional Adult Outcomes 16 Years After Childhood Diagnosis of Attention-Deficit/Hyperactivity Disorder: MTA Results.	Hechtman et al	2016	Ineligible outcome - study primarily assessed functional, occupational, emotional, substance-use and other adult outcomes; deaths were reported descriptively but mortality was not a primary or formally analysed outcome.
132. Long-term outcomes of pharmacologically treated versus non-treated adults with ADHD and substance use disorder: a naturalistic study.	Bihlar muld et al	2015	ADHD not main predictor - study population consisted exclusively of adults with ADHD and severe comorbid substance use disorder receiving compulsory treatment; therefore, findings are not representative of the general ADHD population.

© 2026 Corrigan L et al. *JAMA Network Open*.

133. Traffic and Criminal Behavior of Adults with Attention Deficit-Hyperactivity with a Prospective Follow-Up from Birth to the Age of 40 Years.	Koisaari et al	2015	Ineligible outcome - mortality was only descriptively reported and was not the primary outcome of the study.
134. Cardiovascular safety of central nervous system stimulants in children and adolescents: population based cohort study.	Winterstein et al	2012	Ineligible population - study included children with ADHD and other mental health conditions (e.g., adjustment reaction, conduct disturbance and emotional disturbance), rather than an ADHD-specific population. Also the stimulant users were drawn from a broader population of children with ADHD and other mental health conditions; the study was not restricted to individuals with ADHD.
135. ADHD medications and risk of serious cardiovascular events in young and middle-aged adults.	Habel et al	2011	Ineligible population and outcome - the cohort was defined by ADHD medication use rather than confirmed ADHD diagnosis; therefore it was not an ADHD-defined population for assessing mortality associated with ADHD. Also, the study assessed a composite cardiovascular outcome (MI, stroke and sudden

© 2026 Corrigan L et al. *JAMA Network Open*.

			cardiac death), rather than standalone all-cause mortality.
136. ADHD drugs and serious cardiovascular events in children and young adults.	Cooper et al	2011	Ineligible outcome – This study primarily assessed a composite of serious cardiovascular events (sudden cardiac death, myocardial infarction and stroke), rather than mortality as a standalone outcome.
137. Cardiac safety of central nervous system stimulants in children and adolescents with attention-deficit/hyperactivity disorder.	Winterstein et al	2007	Ineligible outcome - study evaluated cardiac death and other cardiac events in relation to stimulant exposure, rather than all-cause mortality associated with ADHD medication.
138. ADHD Pharmacotherapy and Mortality in Individuals with ADHD: A Target Trial Emulation Study with Observational Data	Chang et al	2024	Duplicate
139. Sudden Death and Use of Stimulant Medications in Youths	Gould et al	2009	Ineligible population – this study included youths with sudden unexplained death and did not require an ADHD diagnosis; ADHD status could not be reliably determined.
140. Mortality associated with ADHD drug treatment	McCarthy et al	2007	Ineligible population -cohort was defined by ADHD medication prescribing rather than a confirmed ADHD diagnosis/ identification/ trait measure;

© 2026 Corrigan L et al. *JAMA Network Open*.

			therefore the study population was not restricted to individuals with ADHD.
141. Attention deficit hyperactivity disorder associated with increased mortality	Zehetner	2015	Ineligible publication type - commentary/summary of an original mortality study; does not provide original study data.
142. The relationship of ADHD and trauma mortality: An NTDB analysis	Zwemer et al	2024	Insufficient statistical information
143. Methylphenidate and Risk of Serious Cardiovascular Events in Adults	Schelleman et al	2012	Ineligible population -cohort was defined by ADHD medication prescribing rather than a confirmed ADHD diagnosis/ identification/ trait measure; therefore the study population was not restricted to individuals with ADHD.
144. Comparing Childhood Characteristics of Adopted and Non-adopted Individuals Deceased by Suicide.	Ligier et al	2022	Ineligible outcome - suicide death was the case-selection criterion; ADHD was assessed as a characteristic rather than as a predictor of mortality.
145. Increased risk of COVID-19 infection and mortality in people with mental disorders: analysis from electronic health records in the United States.	Wang et al 2021	2021	ADHD not main predictor - study evaluated COVID-19 infection and mortality associated with multiple mental disorders; ADHD was one of several psychiatric exposures and was not specifically analysed

© 2026 Corrigan L et al. *JAMA Network Open*.

			as the primary predictor of mortality.
146. Contribution of developmental disabilities to childhood mortality in the United States: a multiple-cause-of-death analysis.	Boyle et al	1994	ADHD not main predictor - study examined mortality associated with developmental disabilities as a broad group; ADHD was one of several developmental disabilities included rather than the primary exposure.
147. Rising Rate of Hospitalizations in Adolescents with Attention Deficit Hyperactive Disorder in Spain.	Blasco-fontecilla et al	2025	Insufficient statistical information
148. Association of ADHD symptoms in adolescence and mortality in Northern Finland Birth Cohort 1986.	Mustonen et al	2023	Ineligible predictor - study examined dimensional ADHD symptoms in a population-based cohort rather than mortality associated with a clinical ADHD diagnosis/ self-identified ADHD. Also this study used a general-population birth cohort; participants were not selected based on an ADHD diagnosis.
149. Risk factors for pre-adolescent onset suicidal behavior in a high-risk sample of youth.	Zelazny	2021	Ineligible outcome - study examined suicidal behaviour, including suicide attempts and severe suicidal ideation, rather than mortality/death.

© 2026 Corrigan L et al. *JAMA Network Open*.

eFigure 3: Funnel plot for all-cause mortality

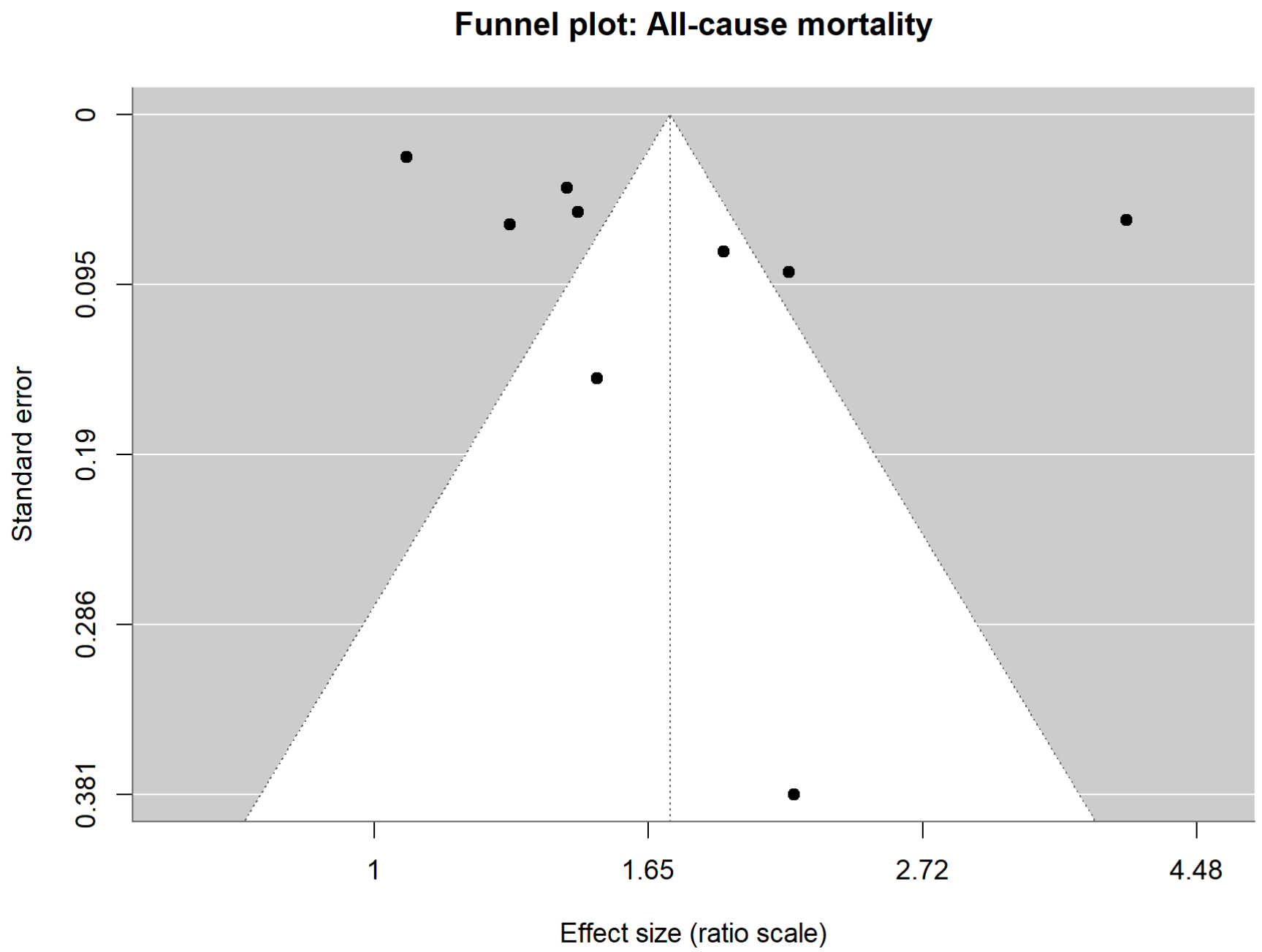

© 2026 Corrigan L et al. *JAMA Network Open*.

eFigure 4: Funnel plot for suicide mortality

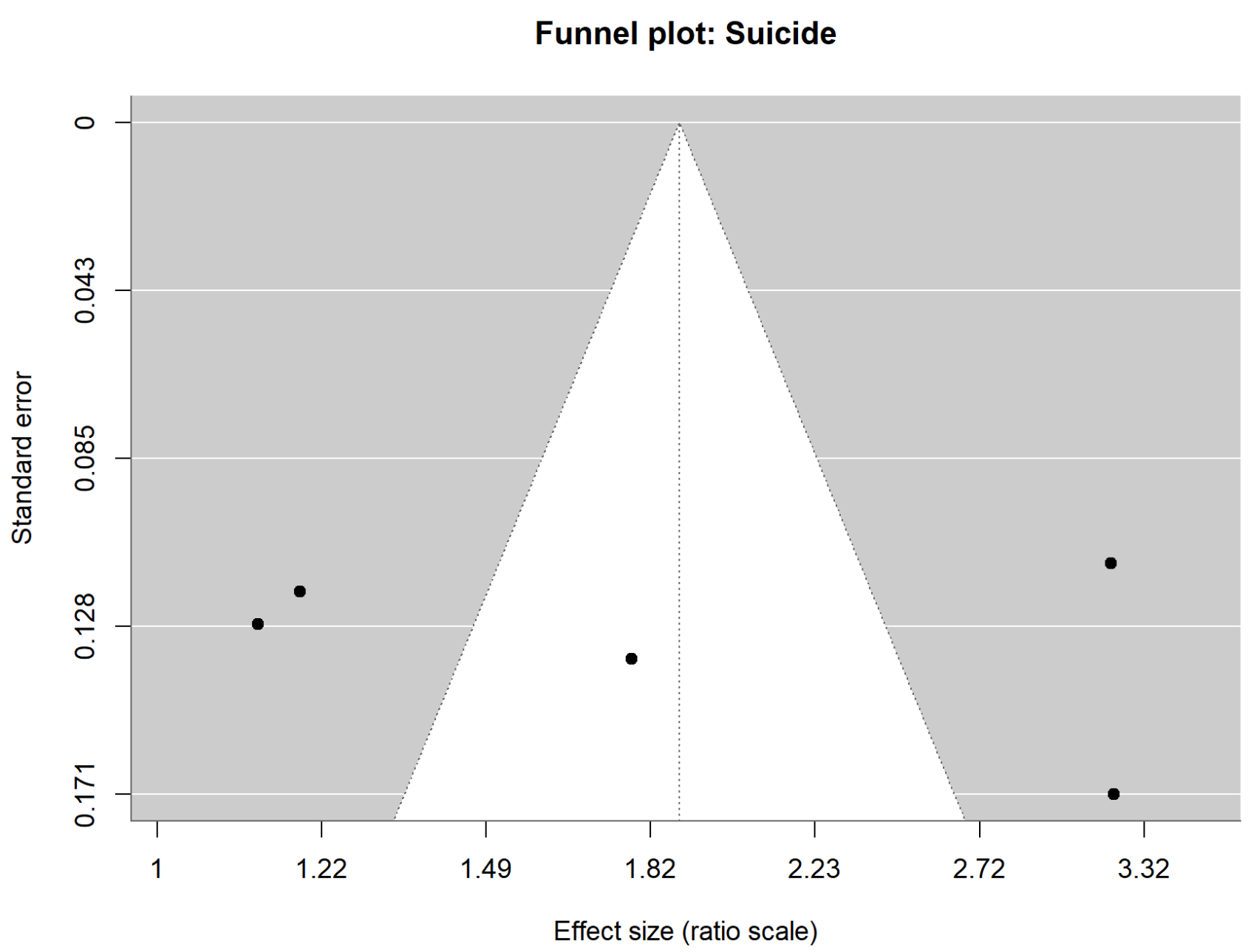

© 2026 Corrigan L et al. *JAMA Network Open*.

eFigure 5: Funnel plot for accident or injury deaths

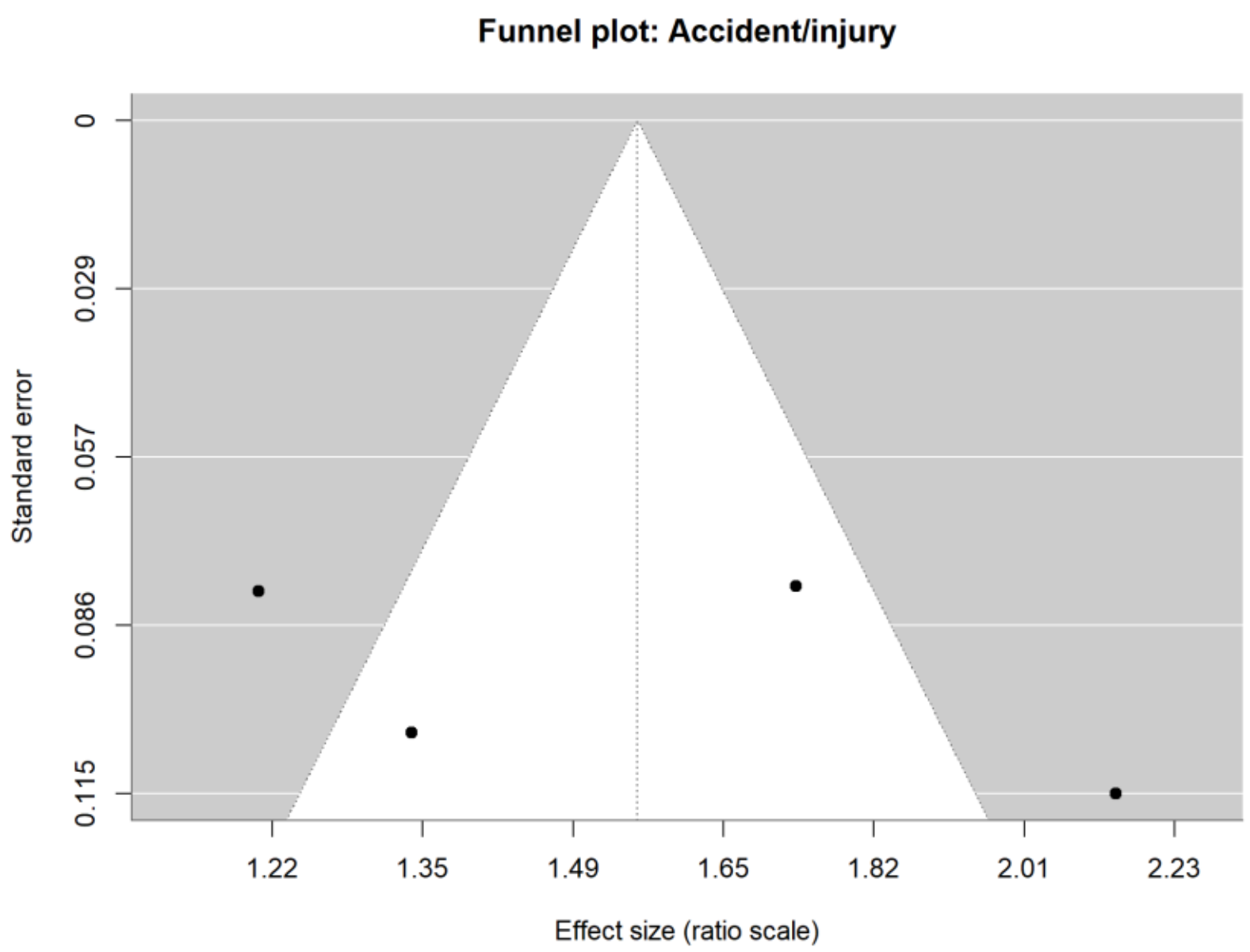

© 2026 Corrigan L et al. *JAMA Network Open*.

eFigure 6: Funnel plot for natural cause of death

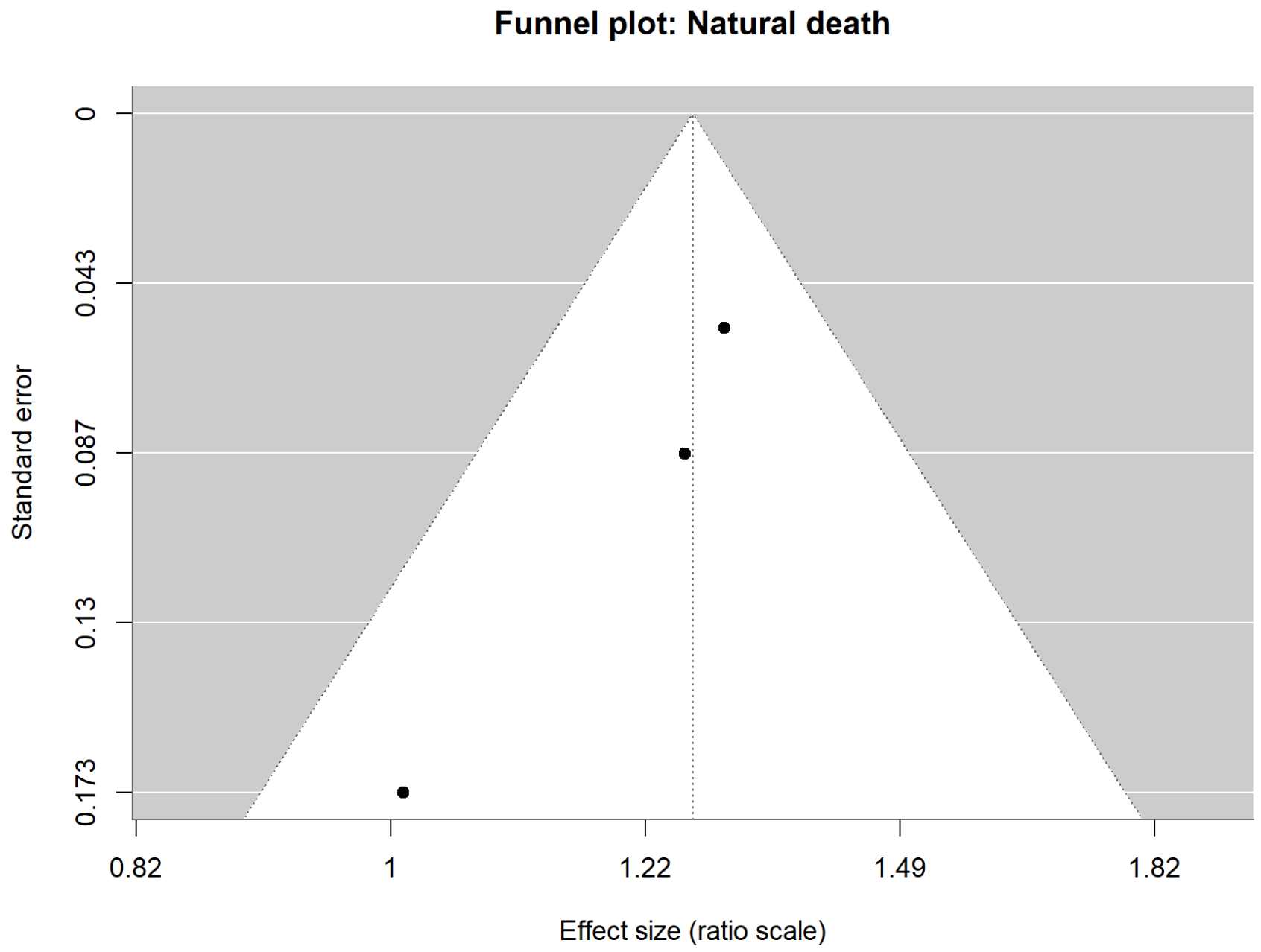

© 2026 Corrigan L et al. *JAMA Network Open*.

eFigure 7: Funnel plot for all-cause mortality in medication use

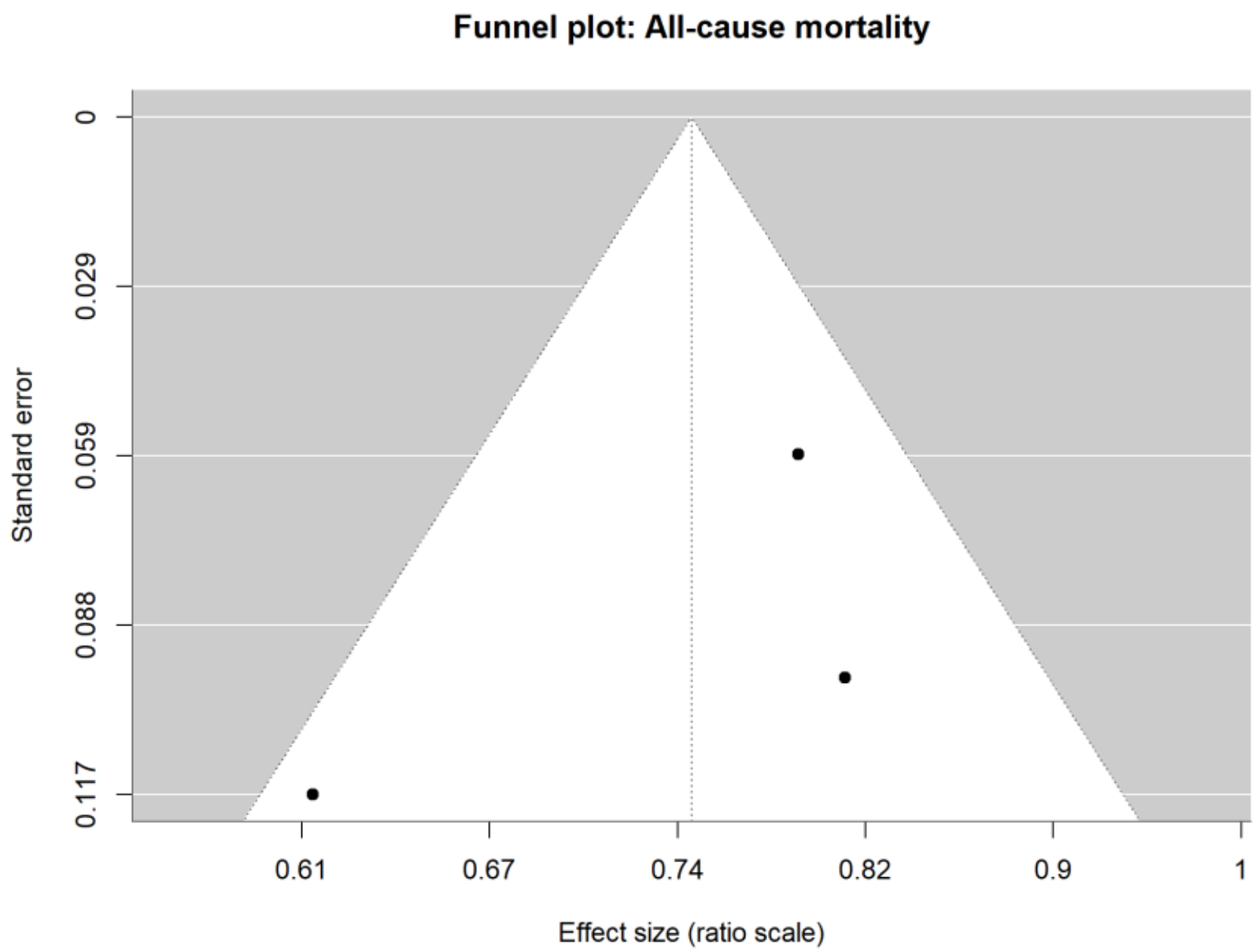

© 2026 Corrigan L et al. *JAMA Network Open*.

eFigure 8: Sensitivity analysis forest plot

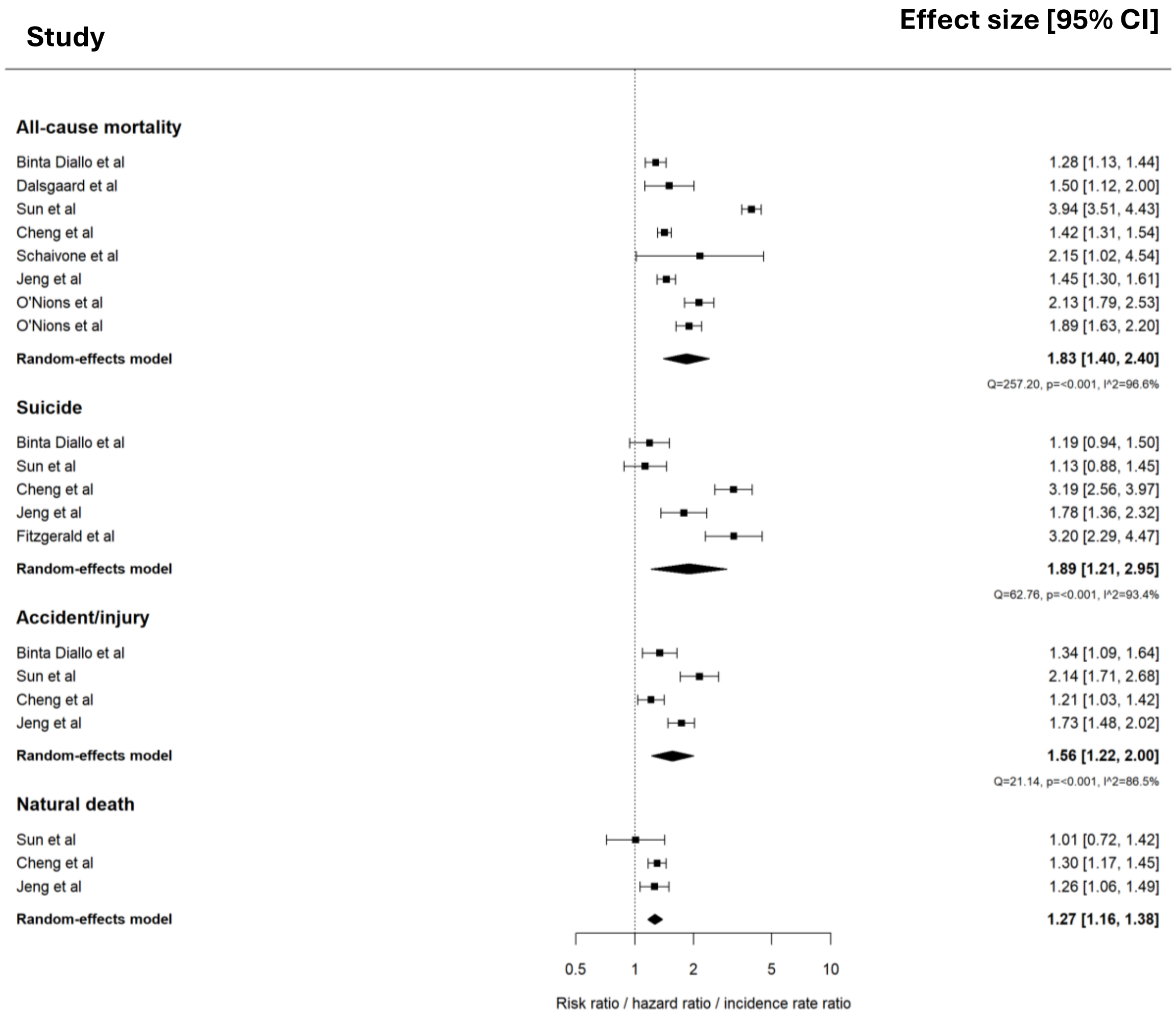

© 2026 Corrigan L et al. *JAMA Network Open*.

eFigure 9: Sensitivity analysis: Funnel plot

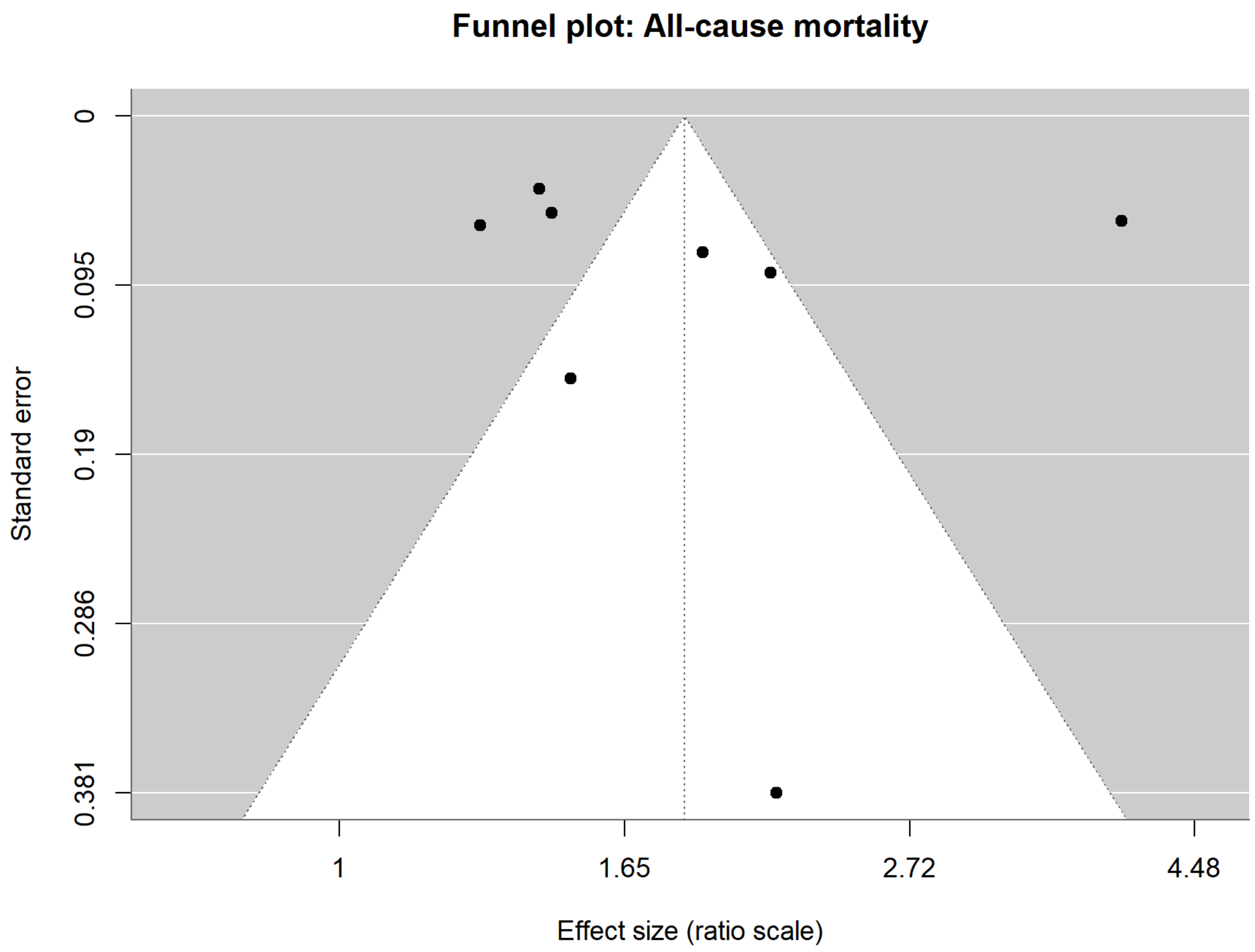

© 2026 Corrigan L et al. *JAMA Network Open*.
