# Supplementary material for: Mortality Among People With Attention-Deficit/Hyperactivity Disorder: A Systematic Review and Meta-Analysis
Source: JAMA Netw Open. 2026 Oct 19;9(10):e2639448. doi: 10.1001/jamanetworkopen.2026.39448 (PMC13681224; doi:10.1001/jamanetworkopen.2026.39448)
Supplement: Supplementary file 2 [file jamanetwopen-e2639448-s002.pdf]

Data Sharing Statement

Corrigan. Mortality Among People With ADHD. *JAMA Netw Open*. Published October 19, 2026. doi:10.1001/jamanetworkopen.2026.39448

Data
Data available: Yes
Data types: Data (not involving human participants)
How to access data: Data can be requested from Author (lucycorr@liverpool.ac.uk)
When available: With publication

Supporting Documents
Document types: None

Additional Information
Who can access the data: anyone requesting data
Types of analyses: Meta-analyses
Mechanisms of data availability: Data can be requested from Author (lucycorr@liverpool.ac.uk)
